# Incorporating early cfEBV DNA clearance into clinical risk stratification to tailor induction chemotherapy cycles for locoregionally advanced nasopharyngeal carcinoma

**DOI:** 10.1038/s41416-026-03401-5

**Published:** 2026-03-31

**Authors:** Wan-Ping Guo, Xuan Yu, Zi-Jian Lu, Yu-Chen Li, Chun Wu, Li-Wen Gu, Jing-Na Cao, Dong-Hua Luo, Sai-Lan Liu, Ling Guo

**Affiliations:** 1 Department of Nasopharyngeal Carcinoma, Sun Yat-sen University Cancer Center, State Key Laboratory of Oncology in South China, Collaborative Innovation Center for Cancer Medicine, Guangdong Key Laboratory of Nasopharyngeal Carcinoma Diagnosis and Therapy, Guangdong Provincial Clinical Research Center for Cancer, Sun Yat-sen University Cancer Center, Guangzhou, PR China; 2 Department of Intensive Care Unit, Sun Yat-sen University Cancer Center, State Key Laboratory of Oncology in South China, Collaborative Innovation Center for Cancer Medicine, Guangdong Key Laboratory of Nasopharyngeal Carcinoma Diagnosis and Therapy, Guangdong Provincial Clinical Research Center for Cancer, Sun Yat-sen University Cancer Center, Guangzhou, PR China

**Keywords:** Tumour biomarkers, Chemotherapy

## Abstract

**Background:**

The optimal number of induction chemotherapy (IC) cycles for locoregionally advanced nasopharyngeal carcinoma (LA-NPC) is debated. This study investigates if early cell-free Epstein-Barr virus (cfEBV) DNA clearance can personalise treatment.

**Methods:**

We included 1590 LA-NPC patients treated with IC+ chemoradiotherapy (2010–2023) with complete early cfEBV DNA data. COX regression identified independent prognostic factors, and receiver operating characteristic curves assessed predictive accuracy. Propensity score matching (PSM) balanced covariates between groups receiving different IC cycles. The primary outcome, progression-free survival (PFS) was analysed using Kaplan-Meier and log-rank tests.

**Results:**

After the first IC cycle, 45.5% of patients had undetectable cfEBV DNA. Combining cfEBV DNA clearance, N-stage, and overall stage yielded the highest AUC for 5-year PFS (0.632). Patients were stratified into high- and low-risk groups (*p* < 0.001). Post-matching, low-risk patients receiving three IC cycles had better 5-year PFS than those receiving two (85.2% vs. 76.0%, *p* = 0.024), with no significant increase in grade 3–4 toxicities (30.36% vs. 24.29%, *p* = 0.157). High-risk patients showed no PFS benefit from additional cycles (63.2% vs. 61.0%, *p* = 0.960).

**Discussion:**

Early cfEBV DNA clearance predicts LA-NPC outcomes. Low-risk patients may benefit from an additional cycle of IC, while high-risk patients may require alternative strategies like immunotherapy or earlier chemoradiotherapy.

## Introduction

Nasopharyngeal carcinoma (NPC), a malignancy arising from the nasopharyngeal epithelium, demonstrates distinct geographical clustering, with over 70% of the global cases concentrated in East and Southeast Asia [1, 2]. Clinically, NPC is characterised by an insidious onset, leading to delayed diagnosis. Consequently, approximately 60–70% of patients present with locoregionally advanced disease (LA-NPC; T3-4/N1–3/M0) at initial diagnosis [3]. Platinum-based concurrent chemoradiotherapy (CCRT) is the therapeutic cornerstone for LA-NPC, which leverages chemotherapy-induced radio-sensitisation to enhance local control [4–6]. However, distant metastasis persists as the predominant cause of treatment failure, accounting for 70% of cancer-specific mortality [7–9]. Therefore, induction chemotherapy (IC) prior to CCRT has been adopted to address micro-metastatic dissemination, with landmark Phase III trials demonstrating significant survival benefits [10–12]. IC followed by CCRT achieved absolute 5-year survival increments of 7.9–9.1% for overall survival (OS) [10–12], securing its position as a Category 1A recommendation in the National Comprehensive Cancer Network (NCCN) and Chinese Society of Clinical Oncology (CSCO) guidelines [13, 14].

Despite these advances, the optimal number of IC cycles remains unknown. Current protocols variably employ 2-4 cycles [11, 15, 16], whereas escalating cycles may paradoxically compromise outcomes. Additionally, prolonged IC administration increases the risk of radiotherapy delay, potentially accelerating tumour repopulation and compromising radiotherapy tolerance due to cumulative toxicities, a therapeutic postponement consistently associated with survival detriment [10, 17–19]. This underscores the need for personalised IC cycle selection.

Moreover, accurate prognostic stratification is pivotal for optimising therapeutic strategies. While TNM staging provides a foundational framework, plasma cell-free Epstein-Barr virus DNA (cfEBV DNA) has emerged as a validated biomarker for NPC [20, 21]. Consequently, pretreatment cfEBV DNA levels, reflecting tumour burden, are frequently integrated with TNM staging for risk stratification or guide IC cycle selection [22–24]. Nonetheless, static baseline measurements fail to capture dynamic treatment responses. Furthermore, although serial imaging offers insights into tumour volume changes, frequent radiographic assessments impose logistic and financial burdens [25]. In this regard, recent evidence suggests that dynamic cfEBV DNA kinetics during therapy may predict outcomes and elucidate tumour biology [26–28], offering a cost-effective alternative to imaging [29]. However, early-phase cfEBV DNA changes (e.g., post-first IC cycle) remain underexplored as predictive tools for IC cycle optimisation in LA-NPC.

Therefore, this study investigated the early dynamic cfEBV DNA changes (pretreatment and post-first IC cycle) in patients with LA-NPC receiving 2–3 IC cycles followed by CCRT, with or without adjuvant therapy (AT). We aimed to establish a biomarker-driven framework for personalised IC cycle selection by correlating these kinetics with survival outcomes, ultimately refining risk-adapted therapeutic strategies in this high-risk population.

## Materials and methods

### Patient selection

This retrospective study enroled consecutive patients with LA-NPC treated at Sun Yat-sen University Cancer Center (SYSUCC) between January 2013 and December 2023. The inclusion criteria were defined as follows: (1) histopathologically confirmed non-keratinising NPC classified as stage III–IVa (8th edition American Joint Committee on Cancer/Union for International Cancer Control TNM stage system) [30]; (2) completion of 2–3 cycles of platinum-based IC followed by definitive intensity-modulated radiotherapy (IMRT) with concurrent platinum-based chemotherapy (CCRT), and adjuvant chemotherapy permitted at clinicians’ discretion; (3) availability of pre- and post-first IC cycle plasma cfEBV DNA quantitation and pretreatment EBV DNA > 0 copies/mL; (4) absence of pregnancy, lactation, or history of synchronous/metachronous malignancies; and (5) preserved organ function (hematopoietic, hepatic, and renal). The patient enrolment workflow is summarised in eFig. 1.

All participants underwent standardised pretreatment evaluations, including comprehensive physical examination, EBV serological profiling, electrocardiography, nasopharyngeal fiberoptic endoscopy with biopsy, contrast-enhanced magnetic resonance imaging (MRI) of the nasopharynx and neck, chest/abdominal contrast-enhanced computed tomography (CT), and bone scan, or whole-body fluorodeoxyglucose positron emission tomography to exclude distant metastases.

### Treatment

All patients included in this study received 2 or 3 cycles of IC combined with CCRT, with or without AC. The common IC regimens included: a) GP, consisting of gemcitabine (1 g/m² on days 1 and 8) and cisplatin (80 mg/m² on day 1); b) TPF, comprising docetaxel (60 mg/m² on day 1), cisplatin (60 mg/m² on day 1), and 5-fluorouracil (500–600 mg/m² on days 1–5); c) TP, consisting of docetaxel (75 mg/m² on day 1) and cisplatin (75 mg/m² on day 1); d) PF, consisting of 5-fluorouracil (800–1000 mg/m², 120-h continuous infusion) and cisplatin (75 mg/m² on day 1); e) TPC, comprising docetaxel (60 mg/m² on day 1), cisplatin (60 mg/m² on day 1), and capecitabine (1000 mg/m², twice daily, days 1–14). IC was administered at 3-week intervals. All patients received radical radiotherapy using IMRT techniques. Target volume delineation and dose constraints for organs at risk (OARs) were performed according to our institutional protocol [7, 31]. The CCRT regimens were platinum-based, primarily using cisplatin. Common agents and dosing regimens included: cisplatin/nedaplatin (80–100 mg/m², day 1), carboplatin (400 mg/m², day 1), or loplatin (80–100 mg/m², day 1), administered every 3 weeks for a total of 2–3 cycles. The AC regimens in this study primarily used capecitabine (1000 mg/m², BID, days 1–14) or S-1 (40–60 mg, BID, days 1–14), administered every 3 weeks, with at least one cycle completed within 3 months after CCRT.

### Plasma cfEBV DNA quantification

Plasma cfEBV DNA levels were analysed via real-time quantitative polymerase chain reaction (qPCR) targeting the BamHI-W genomic region, according to a previously validated protocol [32, 33]. Specifically, plasma samples were collected in ethylenediaminetetraacetic acid (EDTA)-anticoagulant tubes, with 5 mL of peripheral blood obtained from each participant. The blood samples were centrifuged at 1600 × g to separate plasma, which was then carefully aliquoted into polypropylene tubes and stored at -80 °C until subsequent processing. Genomic DNA extraction from plasma specimens was performed using the QIAamp Blood Kit (Qiagen, Hilden, Germany) in accordance with the manufacturer’s blood and body fluid extraction protocol. For each extraction column, 500–1000 μL of plasma was used, and the extracted DNA was eluted in a final volume of 50 μL; the exact volume of plasma input and eluate was recorded for the subsequent calculation of EBV DNA copy concentration. For real-time quantitative polymerase chain reaction (qPCR) targeting EBV DNA, the BamHI-W region of the EBV genome was selected as the detection locus, a well-validated target for EBV DNA quantification in nasopharyngeal carcinoma. The qPCR assay was established with specific amplification primers: W-44F (5’-AGT CTC TGC CTC CAG GCA-3’) and W-119R (5’-ACA GAG GGC CTG TCC ACC G-3’), alongside a dual-labelled fluorescent probe W-67T (5’-[FAM] CAC TGT CTG TAA AGT CCA GCC TCC [TAMRA]-3’). To verify the amplifiability of plasma DNA and exclude extraction or PCR inhibition, the β-actin gene was used as an internal control. The β-actin primers were designed as forward: 5’-ACA GGC ACCA GGG CGT GAT GG-3’ and reverse: 5’-CTC CAT GTC GTC CCA GTT GGT-3’, with a dual-labelled probe (5’-[FAM] CAT CCT CAC CCT GAA GTA CCC CAT C [TAMRA]-3’) [34–36]. Longitudinal monitoring of viral load dynamics was systematically implemented across four critical phases, as described in a previous study [37]: (1) baseline assessment conducted 2 weeks prior to IC initiation; (2) post-first IC cfEBV DNA levels collected within 1 week before 2nd IC cycle; (3) post-IC levels assessed within 1 week before the initiation of CCRT; and (4) post-treatment levels assessed within 1 week upon CCRT completion. The cutoff value for pre-treatment cfEBV DNA was defined as 4000 copies/ml, consistent with our previously validated clinical threshold [12, 38–41].

### Follow-up and outcomes

Following the completion of CCRT, patients were typically instructed to undergo scheduled follow-up assessments quarterly during the first 2 years, semi-annually for the subsequent 3 years, and annually thereafter until death. Standard surveillance incorporated nasopharyngoscopy, head-neck contrast-enhanced MRI, chest X-ray or CT, abdominal ultrasound or CT, and plasma cfEBV DNA quantification.

The primary endpoint was progression-free survival (PFS), calculated from treatment initiation to disease progression, death from any cause, or censoring at the last follow-up. Secondary endpoints comprised OS, calculated from the time of treatment initiation to death. Distant metastasis-free survival (DMFS) as the duration until first distant metastasis or death, and locoregional recurrence-free survival (LRRFS) as the interval until initial locoregional recurrence or death. Censoring was applied at the final documented follow-up for event-free patients. Acute adverse events during IC were graded according to the Common Terminology Criteria for Adverse Events (Version 5.1).

### Statistical analysis

All statistical analyses were conducted using SPSS 26.0 (IBM Corp.) and R 4.3.2 (R Foundation). Categorical variables were analysed using χ² or Fisher’s exact tests, with continuity correction applied where appropriate. Survival analyses employed Kaplan–Meier methodology with log-rank testing for between-group comparisons. Propensity score matching (PSM) (1:1 ratio) was implemented using the nearest neighbour algorithm (caliper = 0.1 SD) to mitigate selection bias between the 2-cycle and 3-cycle IC groups. The matching covariates encompassed: demographic parameters (age, sex), disease characteristics (T/N/overall stage, KPS), virological profiles (pre-treatment, post-first IC/post-IC/post-CCRT cfEBV DNA), and treatment details (IC regimen, CCT cycles, AT administration).

Multivariate Cox proportional hazards regression models were applied to identify independent prognostic factors. The resulting regression coefficients were used to calculate individual prognostic risk scores. The predictive accuracy of the prognostic model was further evaluated using receiver operating characteristic (ROC) curve analysis, with area under the curve (AUC) values calculated at 5-year timepoints to quantify discrimination ability. Based on the median value of these risk scores, patients were classified into high-risk and low-risk subgroups. All statistical tests were two-sided, with *p* < 0.05 being considered statistically significant.

## Results

### Patient characteristics

After rigorous screening, we identified 1590 patients with LA-NPC from a total of 79,899 newly diagnosed NPC cases who had received 2–3 cycles of IC followed by CCRT and had complete cfEBV DNA data (eFig. 1, Table 1). Analysis of demographic and clinical characteristics revealed a median age of 46 (range: 18–76) years with a significant male predominance (1184 patients, 74.5%). Stage III disease was present in 1107 cases (69.6%). The cohort demonstrated balanced distribution of IC cycles with 857 patients (53.9%) receiving 2 cycles and 733 (46.1%) patients receiving 3 cycles. Subsequent CCRT cycles followed a similar pattern, with 972 patients (61.1%) completing 2 cycles and 618 (38.9%) patients undergoing 3 cycles. Post-CCRT AT was administered to 173 patients (10.9%). Pretreatment cfEBV DNA analysis identified 612 patients (38.5%) with elevated viral load ≥4000 copies/mL. All patients had positive pretreatment cfEBV DNA; 45.5% of patients achieved undetectable cfEBV DNA after the first IC cycle, 67.5% had cleared cfEBV DNA before radiotherapy, and the proportion reached 94.2% at the end of CCRT (eFig. 2).

**Table 1:** Baseline characteristics before and after PSM matched of the whole cohort.

	Unmatched Cohort			PSM-matched Cohort^a^		
Characteristics^b^	2-Cycle	3-Cycle	*p* value	2-Cycle	3-Cycle	*P* value^c^
Overall	857	733		491	491	
Age (%)			0.102			1.00
≤45	387 (45.2)	362 (49.4)		245 (49.9)	246 (50.1)	
>45	470 (54.8)	371 (50.6)		246 (50.1)	245 (49.9)	
Sex (%)			0.433			1.00
Male	630 (73.5)	554 (75.6)		364 (74.1)	367 (74.7)	
Female	227 (26.5)	179 (24.4)		127 (25.9)	124 (25.3)	
T Stage (%)			0.24			1.00
T1-2	73 (8.5)	76 (10.4)		52 (10.6)	51 (10.4)	
T3-4	784 (91.5)	657 (89.6)		439 (89.4)	440 (89.6)	
N Stage (%)			<0.001			0.698
N0-1	255 (29.8)	59 (8.0)		63 (12.8)	58 (11.8)	
N2-3	602 (70.2)	674 (92.0)		428 (87.2)	433 (88.2)	
Overall stage (%)			<0.001			0.589
III	637 (74.3)	470 (64.1)		330 (67.2)	321 (65.4)	
IVA	220 (25.7)	263 (35.9)		161 (32.8)	170 (34.6)	
ECOG PS (%)			0.006			0.585
0–1	825 (96.3)	682 (93.0)		465 (94.7)	460 (93.7)	
> 1	32 (3.7)	51 (7.0)		26 (5.3)	31 (6.3)	
Induction chemotherapy regimen (%)			<0.001			0.792
GP	119 (13.9)	295 (40.2)		91 (18.5)	86 (17.5)	
TPF	363 (42.4)	213 (29.1)		180 (36.7)	196 (39.9)	
TP	213 (24.9)	115 (15.7)		120 (24.4)	110 (22.4)	
TPC	130 (15.2)	89 (12.1)		77 (15.7)	80 (16.3)	
Other or more	32 (3.7)	21 (2.9)		23 (4.7)	19 (3.9)	
Pre-treatment EBV DNA level (%)			<0.001			1
<4000 copies/mL	592 (69.1)	386 (52.7)		282 (57.4)	283 (57.6)	
≥ 4000 copies/mL	265 (30.9)	347 (47.3)		209 (42.6)	208 (42.4)	
EBV DNA level after first cycle (%)			0.706			0.898
Undetectable	386 (45.0)	338 (46.1)		229 (46.6)	232 (47.3)	
Detectable	471 (55.0)	395 (53.9)		262 (53.4)	259 (52.7)	
EBV DNA level after IC (%)			<0.001			0.733
Undetectable	541 (63.1)	532 (72.6)		330 (67.2)	336 (68.4)	
Detectable	316 (36.9)	201 (27.4)		161 (32.8)	155 (31.6)	
EBV DNA level after CCRT (%)			0.399			0.885
Undetectable	803 (93.7)	695 (94.8)		465 (94.7)	467 (95.1)	
Detectable	54 (6.3)	38 (5.2)		26 (5.3)	24 (4.9)	
Concurrent chemotherapy cycles (%)			<0.001			0.43
2 Cycles	478 (55.8)	494 (67.4)		311 (63.3)	298 (60.7)	
3 Cycles	379 (44.2)	239 (32.6)		180 (36.7)	193 (39.3)	
Adjuvant therapy (%)			0.002			0.598
No	744 (86.8)	673 (91.8)		438 (89.2)	444 (90.4)	
Yes	113 (13.2)	60 (8.2)		53 (10.8)	47 (9.6)	

*PSM* propensity score matching, *ECOG* *PS* Eastern Cooperative Oncology Group performance status, *T* tumour, *N* node, *EBV* Epstein‑Barr virus, *IC* Induction chemotherapy, *CCRT* Concurrent chemoradiotherapy, *GP* Gemcitabine plus cisplatin, *TPF* Docetaxel plus cisplatin plus fluorouracil, *TP* Docetaxel plus cisplatin, *TPC* Docetaxel plus cisplatin plus capecitabine.

^a^PSM was conducted using the 1:1 nearest-neighbour matching method to balance baseline characteristics between the 2-cycle and 3-cycle induction chemotherapy groups;

^b^Categorical variables are presented as *n* (%);

^c^*p* value was calculated by chi-squared test, or Fisher’s exact test, as appropriate.

### Outcomes of different cycles in the whole cohort

During a median follow-up duration of 33.1 months (IQR: 21.37–51.33), the 3- and 5-year PFS rates for the whole cohort were 81.3% and 74.1%, respectively. Survival outcomes in the entire cohort were analysed based on IC cycles. Before PSM, patients receiving 3-cycle IC showed no significant improvement in any survival outcome (all *p* > 0.05) compared to those receiving 2-cycle IC (eFig. 3A, C, E, G). Subgroup analyses revealed that patients with higher pre-treatment cfEBV DNA levels who received three IC cycles achieved a borderline significant PFS advantage (*p* = 0.054). In contrast, other early-treatment characteristics did not show significant differences in outcomes after two or three IC cycles (all *p* > 0.05), as shown in eFig. 4A.

PSM resulted in 491 patients in each group. Patients who received 3-cycle IC had significantly better PFS (HR = 0.72, 95% CI: 0.55–0.96, *p* = 0.023; eFig. 3B), and the same in OS and DMFS (all *p* < 0.05, details shown in eFig. 3D, F). Notably, LRRFS did not show significant difference between the different IC-cycle groups (*p* = 0.11; eFig. 3H). Subgroup analyses demonstrated that among patients with a higher N stage (*p* = 0.023), an advanced overall stage (*p* = 0.018), or higher pre-treatment cfEBV DNA levels (*p* = 0.007), those who received 3-cycle IC experienced a notable improvement in PFS. Specifically, patients who achieved cfEBV DNA clearance after the first cycle of IC also showed a more favourable PFS outcome when they underwent 3-cycle IC (*p* = 0.007). Additional subgroup analyses details are shown in eFig. 4B.

### Prognosis values of post-first IC cfEBV DNA in the whole cohort

Both univariate and multivariate Cox regression analyses (Table 2) verified that a higher N stage (*p* = 0.008), an advanced overall stage (*p* = 0.030), and persistent cfEBV DNA after the first IC cycle (*p* = 0.014), after all IC cycles (before CCRT, *p* < 0.001), and after CCRT (*p* < 0.001) were negative prognostic factors for the PFS of the entire cohort.

**Table 2:** Univariate and multivariate COX regression analyses for the whole cohort before PSM.

	Univariate Cox Analysis		Multivariate Cox Analysis	
Characteristics	HR (95% CI)	*P* value	HR (95% CI)	*P* value
Age		0.437		
≤45	Reference			
>45	1.1 (0.87–1.38)			
Sex		0.643		
Male	Reference			
Female	0.94 (0.71–1.23)			
T Stage		0.783		
T1–2	Reference			
T3–4	0.95 (0.65–1.39)			
**N Stage**		**0.003**		**0.008**
N0–1	Reference		Reference	
N2–3	1.78 (1.22–2.58)		1.67(1.14–2.44)	
**Overall stage**		**0.020**		**0.03**
III	Reference		Reference	
IVA	1.33 (1.05–1.7)		1.31 (1.03–1.67)	
ECOG PS		0.261		
0–1	Reference			
>1	1.32 (0.81–2.16)			
Induction chemotherapy regimen				
GP	Reference			
TPF	0.91 (0.66–1.24)	0.535		
TP	1.17 (0.83–1.66)	0.373		
TPC	1.05 (0.68–1.63)	0.816		
Other or more	1.61 (0.97–2.68)	0.065		
Induction chemotherapy cycle		0.949		
2 Cycles	Reference			
3 Cycles	0.99 (0.79–1.25)			
Pre-treatment EBV DNA level		**<0.001**		0.065
<4000 copies/mL	Reference		Reference	
≥4000 copies/mL	1.62 (1.28–2.04)		1.25 (0.99–1.59)	
**EBV DNA level after first cycle**		**<0.001**		**0.014**
Undetectable	Reference		Reference	
Detectable	2 (1.56–2.57)		1.41 (1.07–1.87)	
EBV DNA level after IC		**<0.001**		**< 0.001**
Undetectable	Reference		Reference	
Detectable	2.23 (1.76–2.81)		1.77 (1.36–2.32)	
EBV DNA level after CCRT		**<0.001**		**< 0.001**
Undetectable	Reference		Reference	
Detectable	3.63 (2.5–5.26)		2.50 (1.69–3.70)	
Concurrent chemotherapy cycles		**<0.001**		**< 0.001**
2 Cycles	Reference		Reference	
3 Cycles	0.61 (0.47–0.8)		0.6 (0.46–0.79)	
Adjuvant therapy		0.834		
No	Reference			
Yes	0.96 (0.64–1.43)			

*PSM* Propensity score matching, *ECOG PS* Eastern Cooperative Oncology Group performance status, *T* tumour, *N* node, *EBV* Epstein‑Barr virus, *IC* Induction chemotherapy, *CCRT* Concurrent chemoradiotherapy, *GP* Gemcitabine plus cisplatin, *TPF* Docetaxel plus cisplatin plus fluorouracil, *TP* Docetaxel plus cisplatin, *TPC* Docetaxel plus cisplatin plus capecitabine.

We selected three early-stage clinical parameters, the N stage, overall stage, and post-first IC cfEBV DNA, which were significantly associated with PFS, to develop a prognostic model and guide IC cycle decisions. ROC curve analysis (eFig. 5A) demonstrated superior discriminative ability for PFS at the 60-month timepoint: the combined model (AUC = 0.632) outperformed single parameters (all *p* < 0.001) and dual-parameter combinations (AUC_N stage+cfEBV DNA_ = 0.617, *p* = 0.029; AUC_overall stage+cfEBV DNA_ = 0.605, *p* = 0.002). This predictive advantage also extended to secondary outcomes (eFig. 5B-D).

### High and low-risk stratification based on prognostic scoring model

Subsequently, we constructed a prognostic scoring model based on the Cox regression coefficients for *N* stage (*β* = 0.5118), overall stage (*β* = 0.2702), and post-first IC cfEBV DNA (*β* = 0.3465), calculated as: *Prognostic Score* = *(0.5118 × N stage)* + *(0.2702 × overall stage)* + *(0.3465 × post-first IC cfEBV DNA)*. Using the median prognostic score of 2.451 (range: 1.669–2.797) as the threshold, patients were stratified into low-risk (≤2.451, *n *= 889) and high-risk (>2.451, *n* = 701) groups (eTable 1). The high-risk group exhibited significantly inferior outcomes compared to the low-risk group (all *p* < 0.05, eFig. 6). Particularly, survival rates at 3- and 5-years demonstrated marked disparities, with PFS rates of 87.3% and 81.4% in the low-risk group versus 73.8% and 65.1% in the high-risk group, respectively. Multivariate Cox analysis confirmed this risk stratification to be an independent prognostic factor for PFS (adjusted HR = 1.60, 95% CI: 1.24–2.07, *p* < 0.001) (eFig. 7).

### Optimal IC cycles for high- and low-risk groups

**Fig. 1:**
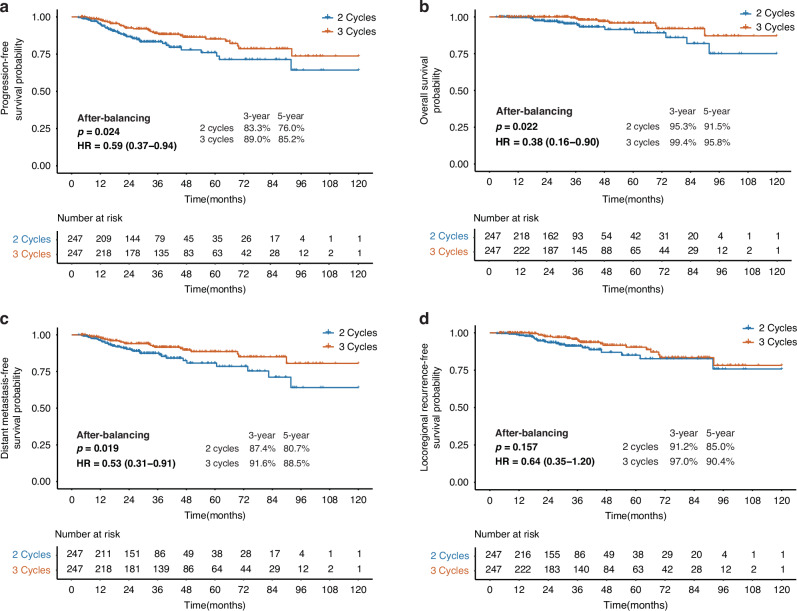
Survival outcomes comparison between 2-cycle and 3-cycle IC of the low-risk group after PSM. **a** Progression-free survival after PSM; **b** Overall survival after PSM; **c** Distant metastasis-free survival after PSM; **d** Locoregional recurrence-free survival after PSM.

Next, we assessed the differential efficacy of IC cycles across risk-stratified cohorts. In the low-risk group, 520 and 369 patients underwent 2-cycle and 3-cycle IC, respectively. While the initial unmatched analysis revealed no survival advantage for extended IC cycles across endpoints (all *p* > 0.05, eFig. 8A-D), an imbalance in baseline prognostic factors was observed (all *p* < 0.05) (eTable 2). Therefore, PSM was performed to address these confounders (*n* = 247 for each group). Post-PSM, the 3-cycle regimen demonstrated clinically meaningful benefits: PFS (HR = 0.59, 95% CI: 0.37–0.94, *p* = 0.024, Fig. 1a), OS (HR = 0.38, 95% CI: 0.16–0.90, *p* = 0.024, Fig. 1b), DMFS (HR = 0.53, 95% CI: 0.31–0.91, *p* = 0.019, Fig. 1c). Meanwhile, LRRFS remained comparable between groups (HR = 0.64, 95% CI: 0.35–1.20, *p* = 0.157, Fig. 1d). Multivariate Cox regression corroborated these findings, confirming a 41% reduction in progression risk with additional IC cycles (*p* = 0.029) within the matched cohort (eTable 3, eFig. 9A).

**Fig. 2:**
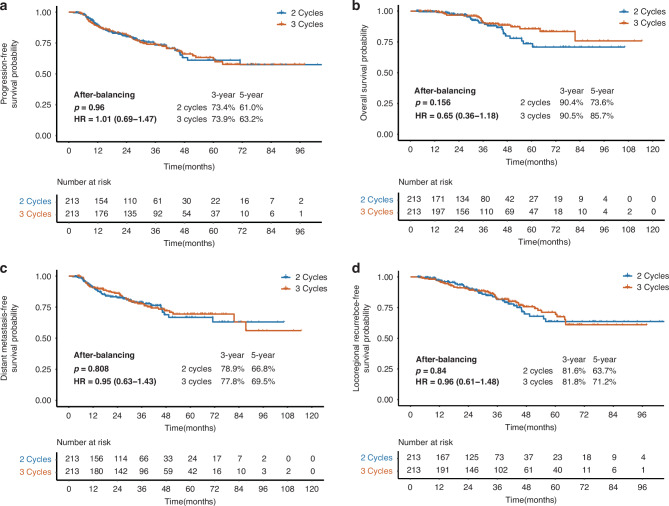
Survival outcomes between 2-cycle and 3-cycle IC of the high-risk group after PSM. **a** Progression-free survival after PSM; **b** Overall survival after PSM; **c** Distant metastasis-free survival after PSM; **d** Locoregional recurrence-free survival after PSM.

In contrast, the high-risk cohort, comprising 337 and 364 patients who underwent 2-cycle and 3-cycle IC, exhibited no differences in survival outcomes (eTable 4). Pre-PSM comparisons showed overlapping survival curves for all outcomes (all *p* > 0.05, eFig. 10A-D). Post-PSM analysis (213 patients for each group) maintained these findings, with comparable PFS (HR = 1.01, 95% CI: 0.69–1.47, *p* = 0.960, Fig. 2a), OS (HR = 0.65, 95% CI: 0.36–1.18, *p* = 0.156, Fig. 2b), DMFS (HR = 0.95, 95% CI: 0.63–1.43, *p *= 0.808, Fig. 2c), and LRRFS (HR = 0.96, 95% CI: 0.61–1.48, *p* = 0.840, Fig. 2d). Cox regression modelling confirmed that the CCT cycles (*p* = 0.041), rather than IC cycles (*p* = 0.984), was the prognostic factor for PFS in the high-risk group (eTable 5, eFig. 9B).

### Adverse events during IC between different IC cycle arms in high- and low-risk groups

No grade 5 adverse events occurred in the PSM-matched low-risk and high-risk cohorts (Table 3). Patients receiving 3-cycle IC showed a numerically higher, but non-significant, incidence of grade 3–4 toxicities compared with those receiving 2-cycle IC (30.36% [75/247] vs. 24.29% [60/247]; *p* = 0.157). Haematologic toxicities were the predominant severe adverse events, with neutropenia showing the highest frequency (3-cycle vs. 2-cycle: 24.7% vs. 18.2%), followed by leukopenia (3-cycle vs. 2-cycle: 11.7% vs. 9.7%). Mild-to-moderate anaemia was the most frequent low-grade toxicity (3-cycle vs. 2-cycle: 66.4% vs. 60.3%).

**Table 3:** Adverse effect of PSM-balancing high- and low-risk groups during induction chemotherapy.

Toxic Effect	Low-risk group				*P* value^c^	High-risk group				P value
	2 cycles (*n* = 247)		3 cycles (*n* = 247)			2 cycles (*n* = 213)		3 cycles (*n* = 213)		
	Grade 1-2^a^	Grade 3-4	Grade 1-2	Grade 3-4		Grade 1-2	Grade 3-4	Grade 1-2	Grade 3-4	
**Any Grade3-4**^**d**^	/	61 (24.7)^b^	/	77 (31.2)	0.133	/	60 (28.2)	/	67 (31.5)	0.525
**Haematologic**										
Leukopenia	72 (29.1)	24 (9.7)	84 (34.0)	29 (11.7)	0.300	58 (27.2)	29 (13.6)	62 (29.1)	30 (14.1)	0.882
Neutropenia	66 (26.7)	45 (18.2)	56 (22.7)	61 (24.7)	0.185	54 (25.4)	47 (22.1)	43 (20.2)	52 (24.4)	0.437
Anaemia	149 (60.3)	2 (0.8)	164 (66.4)	3 (1.2)	0.305	146 (68.5)	2 (0.9)	158 (74.2)	4 (1.9)	0.243
Thrombocytopenia	18 (7.3)	3 (1.2)	31 (12.6)	6 (2.4)	0.081	28 (13.1)	3 (1.4)	32 (15.0)	6 (2.8)	0.496
**Non-Haematologic**										
ALT increased	122 (49.4)	8 (3.2)	114 (46.2)	3 (1.2)	0.199	103 (48.4)	3 (1.4)	95 (44.6)	3 (1.4)	0.737
AST increased	102 (41.3)	8 (3.2)	106 (42.9)	3 (1.2)	0.308	84 (39.4)	5 (2.3)	89 (41.8)	3 (1.4)	0.711
Total bilirubin increased	48 (19.4)	4 (1.6)	49 (19.8)	5 (2.0)	0.936	37 (17.4)	2 (0.9)	40 (18.8)	3 (1.4)	0.834
Creatinine increased	46 (18.6)	1 (0.4)	49 (19.8)	0 (0.0)	0.576	35 (16.4)	0 (0.0)	38 (17.8)	1 (0.5)	0.557
Hypoalbuminemia	116 (47.0)	0 (0.0)	137 (55.5)	0 (0.0)	0.072	105 (49.3)	0 (0.0)	130 (61.0)	1 (0.5)	**0.027**
Hypokalemia	59 (23.9)	1 (0.4)	63 (25.5)	1 (0.4)	0.916	52 (24.4)	0 (0.0)	53 (24.9)	2 (0.9)	0.361
Hyponatremia	101 (40.9)	5 (2.0)	108 (43.7)	5 (2.0)	0.814	78 (36.6)	3 (1.4)	83 (39.0)	2 (0.9)	0.812

*PSM* propensity score matching, *ALT* alanine aminotransferase, *AST* aspartate aminotransferase.

^a^All adverse effects were evaluated according to the CTCAE (Common Terminology Criteria for Adverse Events) Version 5.1, and graded as Grade 1–5. No Grade 5 appears during treatment.

^b^Categorical variables (adverse effect grades) are presented as *n* (%), where *n* represents the number of patients with corresponding adverse effects, and (%) represents the proportion of patients in the respective group.

^c^*p* value was calculated by chi-squared test, or Fisher’s exact test, as appropriate.

^d^The total number and proportion of patients who experienced any Grade 3–4 adverse effect during induction chemotherapy, regardless of the type of toxic effect.

Similarly, in the PSM-matched high-risk group, the incidence of grade 3–4 adverse events was comparable between the 3-cycle (31.5% [67/213]) and 2-cycle (28.2% [60/213]) regimens (*p* = 0.525). Neutropenia was the most common grade 3–4 adverse event (3-cycle vs. 2-cycle: 24.4% vs. 22.1%), while anaemia was the most frequent mild adverse event (3-cycle vs. 2-cycle: 74.2% vs. 68.5%). Notably, none of the adverse events showed significant differences between the 3- and 2-cycle regimens, except for grade 1–2 hypoalbuminemia, which was significantly more frequent in the 3-cycle group (61.0%) than in the 2-cycle group (49.3%; *p* = 0.027).

## Discussion

Our findings demonstrate that integrating post-first IC cfEBV DNA with N stage and overall disease stage enabled effective patient stratification into distinct prognostic cohorts. Particularly, although the low-risk patients derived clinical benefit from three IC cycles without significant toxicity escalation, the high-risk counterparts failed to gain survival advantages from additional chemotherapy cycles, suggesting the necessity for alternative therapeutic strategies in this subpopulation. This differential response pattern underscores the clinical utility of early biomarker-guided treatment intensification while highlighting the biological heterogeneity of chemotherapy-resistant cases.

To the best of our knowledge, our study represents the first systematic integration of post-first IC cfEBV DNA into prognostic stratification and therapeutic decision-making frameworks for LA-NPC. Previous studies have established pretreatment cfEBV DNA as a valuable complement to clinical staging [42, 43] and demonstrated its synergistic prognostic value when combined with clinicopathological parameters [44, 45]. However, existing models relying on baseline cfEBV DNA measurements fail to address two fundamental biological realities, the dynamic clonal evolution during treatment and the need for adaptive therapeutic strategies guided by real-time tumour response [46]. In the present study, we focused on the dynamic change of cfEBV DNA during treatment, and demonstrated that post-first IC cfEBV DNA rather than pre-treatment cfEBV DNA, was the significant prognostic factor for PFS of LA-NPC. This observation aligns with previous study. Lv et al. [37]. pioneered a kinetic profiling approach to stratify patients into distinct prognostic clusters based on longitudinal cfEBV DNA dynamics during treatment. Their analysis revealed that patients achieving immediate clearance after the first IC cycle, termed as “early molecular responders,” exhibited significantly superior disease-free survival compared to non-responders (HR = 2.71, 95% CI: 1.82–4.03, *p* < 0.001), thereby validating the platinum-sensitive phenotype within this subgroup. However, Lv et al. did not construct an exploration for optimal IC cycles in this manuscript.

Although IC is an important component in the treatment of LA-NPC [47–49], our study and previous evidence [50] confirm that the main benefit of IC lies in the improvement of OS/DMFS rather than LRRFS, which may be due to the high locoregional control rate (>90%) achieved within modern IMRT/CCRT [6, 51]. However, the optimal number of cycles remains controversial. Our analysis of 1591 patients revealed clinically meaningful PFS improvements favouring 3-cycle IC, contrasting with prior PSM studies [25, 52] reporting equivalent outcomes between 2-/3-cycle regimens. This discrepancy stems from three distinctions: (1) enhanced statistical power (triple the sample size of prior cohorts); (2) incorporation of longitudinal cfEBV DNA profiling (post-IC/post-CCRT [32, 53]); and (3) enriched representation of stage III patients (66% vs. 36–56% historically), better reflecting LA-NPC’s biological heterogeneity. Although Jiang et al. [54] concluded that 3 cycles of IC could benefit N2-3 patients or responders after 2 cycles, conventional imaging was unable to detect micrometastases leading to systemic progression. Similarly, Lv et al. [26] found that extended IC was ineffective for persistent cfEBV DNA positivity after 2 IC cycles. Our study advances this by utilising cfEBV DNA after the first IC cycle for early decision-making. Post-PSM, low-risk patients (limited tumour burden, platinum-sensitive) derived survival benefits from 3-cycle IC via micrometastasis eradication and tumour volume reduction. In contrast, high-risk patients (bulky disease, chemoresistance) showed no benefit, likely due to delayed RT enabling resistant clones to proliferate [19] and exacerbated toxicity (e.g., higher hypoalbuminemia incidence). Persistent cfEBV DNA after the first IC cycle signals chemoresistance, warranting timely transition to RT or immunotherapy rather than prolonged IC. As Lv et al. demonstrated, RT enhances tumour clearance in non-responders, underscoring its role in overcoming chemoresistance.

In contrast to the study by Lv et al. [37], which determined the administration of 3 or 4 cycles of IC based on whether cfEBV DNA was cleared after 2 cycles of IC, the present study advanced the observation time point to after the first cycle of IC and explored whether patients with LA-NPC in different risk stratifications were more suitable for 2 or 3 cycles of IC. On the one hand, 2–3 cycles of IC are more consistent with clinical practice; most importantly, the earlier time window reduced patients’ waiting time for radiotherapy—a known poor prognostic factor, and alleviated the adverse reactions, economic burden, and psychological stress caused by unnecessary chemotherapy. Second, while Lv et al. [26] merely defined patients with cfEBV DNA clearance after the first cycle of IC as “early responders”, our study achieved clinical translation of this biomarker for the first time. We demonstrated that cfEBV DNA detection after the first cycle of IC, combined with N stage and overall stage (AUC = 0.632), was sufficient to support decision-making regarding IC cycles. Furthermore, the sample size of Lv et al.’s 2019 study was 673 patients, and our sample size was more than twice that of theirs. This not only enhanced the statistical power to validate the value of early cfEBV DNA clearance after the first cycle of IC as a prognostic biomarker but also clarified the efficacy differences between 2 and 3 cycles of IC through rigorous propensity score matching—a key comparison that lacked persuasiveness in previous small-sample studies. In summary, while validating the prognostic value of cfEBV DNA identified in previous studies, the present study proposed an optimised IC strategy that aligns with standard clinical practice, is clinically actionable, and can effectively control toxicity, addressing the key limitations of the aforementioned literature.

Furthermore, our toxicity analysis yielded clinically reassuring findings: low-risk patients receiving 3-cycle IC exhibited adverse event rates comparable to those receiving 2-cycle IC, with grade 3–4 toxicities closely mirroring historical data from randomised clinical trials [10, 11, 55]. This favourable safety profile may be attributed to the preserved integrity of the tumour immune microenvironment in low-risk patients, which could enhance chemosensitivity while mitigating off-target toxicities [56]. In contrast, high-risk patients receiving 3-cycle IC experienced a significantly higher incidence of hypoalbuminemia compared to those receiving 2-cycle IC. This disparity probably results from two mechanisms: (1) accelerated catabolism due to heavy tumour burden and (2) aggravated nitrogen imbalance from prolonged IC exposure [57]. Consequently, these findings underscore the necessity of risk-adapted toxicity monitoring, particularly in patients with persistent post-first IC cfEBV DNA, to optimise treatment efficacy while minimising undue toxicity.

Nonetheless, our study has some limitations that warrant consideration. First, while the qPCR-based assay for EBV DNA quantification is inherently prone to inter-laboratory variability, which may partially limit the universal applicability of some conclusions, this study mitigates such concerns by enroling patients uniformly treated at a single institution (Sun Yat-sen University Cancer Center). Moreover, prior work has demonstrated robust quality control for this assay, with minimal within-run (<10%) and between-day (<20%) variation [34]. Additionally, it is important to acknowledge that harmonising EBV DNA copy number cutoffs across different studies and institutions remains an ongoing effort that requires collaborative advancement from the entire field. Second, the exclusive enrolment of patients from endemic regions may limit the generalisability of our findings to non-endemic populations, where EBV-negative histopathological subtypes are more prevalent. Third, the single-centre design necessitates external validation across diverse ethnic and geographic cohorts to confirm the broader applicability of our prognostic model. Finally, although rigorous PSM was employed to mitigate confounding variables, the inherent selection bias of retrospective observational studies cannot be entirely eliminated.

## Conclusion

This study established a novel paradigm for risk-adapted treatment in LA-NPC, utilising post-first IC cfEBV DNA levels in conjunction with conventional staging. This approach enabled the stratification of patients into distinct prognostic groups, highlighting the potential of tailored IC regimens. While three cycles of IC demonstrated benefit in low-risk patients, high-risk patients showed no significant survival advantage with additional chemotherapy, suggesting the need for alternative treatment strategies. Therefore, future research should explore the role of cfEBV DNA kinetics in the current and emerging era of immunotherapy for LA-NPC, to further refine and personalise treatment approaches.

## Supplementary information

Supplementary Figures and Tables

## Data Availability

The datasets supporting the conclusions of this article are not publicly available to protect patient privacy. Data may be accessed upon reasonable request by contacting the first author Wan-Ping Guo, [guowp@sysucc.org.cn]) or the corresponding author (Ling Guo, [guoling@sysucc.org.cn]), subject to institutional approval and compliance with data protection regulations.
