# Supplementary material for: Incorporating early cfEBV DNA clearance into clinical risk stratification to tailor induction chemotherapy cycles for locoregionally advanced nasopharyngeal carcinoma
Source: Br J Cancer. 2026 Mar 31;134(11):1557–67. doi: 10.1038/s41416-026-03401-5 (PMC13184113; doi:10.1038/s41416-026-03401-5)
Supplement: Supplementary file 1 — Supplementary Figures and Tables [file 41416_2026_3401_MOESM1_ESM.docx]

**Supplementary Tables**

**eTable 1 Baseline characteristics comparable between high- and low-risk groups.**

| Characteristics | | Overall | | | Low-risk | | | High-risk | | | P values | |  |
| --- | --- | --- | --- | --- | --- | --- | --- | --- | --- | --- | --- | --- | --- |
| Overall | | 1591 | | | 889 | | | 701 | | |  | |  |
| Age (%) |  | | |  | | | 0.435 | | |  |  |  |  |
| ≤45 | | 749 (47.11) | | | 427 (48.03) | | | 322 (45.93) | | |  | |  |
| >45 | | 841 (52.89) | | | 462 (51.97) | | | 379 (54.07) | | |  | |  |
| Sex (%) |  | | |  | | | 0.148 | | |  |  |  |  |
| Male | | 1184 (74.47) | | | 649 (73.00) | | | 535 (76.32) | | |  | |  |
| Female | | 406 (25.53) | | | 240 (27.00) | | | 166 (23.68) | | |  | |  |
| T stage (%) |  | | |  | | | 0.017 | | |  |  |  |  |
| T1-2 | | 149 (9.37) | | | 69 (7.76) | | | 80 (11.41) | | |  | |  |
| T3-4 | | 1441 (90.63) | | | 820 (92.24) | | | 621 (88.59) | | |  | |  |
| N stage (%) |  | | |  | | | <0.001 | | |  |  |  |  |
| N0-1 | | 314 (19.75) | | | 314 (35.32) | | | 0 (0.00) | | |  | |  |
| N2-3 | | 1276 (80.25) | | | 575 (64.68) | | | 701 (100.00) | | |  | |  |
| Overall stage (%) |  | | |  | | | 0.246 | | |  |  |  |  |
| III | | 1107 (69.62) | | | 630 (70.87) | | | 477 (68.05) | | |  | |  |
| IVA | | 483 (30.38) | | | 259 (29.13) | | | 224 (31.95) | | |  | |  |
| ECOG PS (%) |  | | |  | | | 0.635 | | |  |  |  |  |
| 0-1 | | 1507 (94.78) | | | 840 (94.49) | | | 667 (95.15) | | |  | |  |
| > 1 | | 83 (5.22) | | | 49 (5.51) | | | 34 (4.85) | | |  | |  |
| Induction chemotherapy cycle (%) | |  | | |  | | |  | | | < 0.001 | |  |
| 2 cycles | | 857 (53.90) | | | 520 (58.49) | | | 337 (48.07) | | |  | |  |
| 3 cycles | | 733 (46.10) | | | 369 (41.51) | | | 364 (51.93) | | |  | |  |
| Induction chemotherapy regimen (%) | | |  | | |  | | |  | | | 0.400 | |
| GP | | 414 (26.04) | | | 228 (25.65) | | | 186 (26.53) | | |  | |  |
| TPF | | 576 (36.23) | | | 331 (37.23) | | | 245 (34.95) | | |  | |  |
| TP | | 328 (20.63) | | | 178 (20.02) | | | 150 (21.40) | | |  | |  |
| TPC | | 219 (13.77) | | | 128 (14.40) | | | 91 (12.98) | | |  | |  |
| Other or more | | 53 (3.33) | | | 24 (2.70) | | | 29 (4.14) | | |  | |  |
| Pre-treatment EBV DNA level (%) | | |  | | |  | | |  | | | <0.001 | |
| < 4000 copies/mL | | 978 (61.51) | | | 635 (71.43) | | | 343 (48.93) | | |  | |  |
| ≥ 4000 copies/mL | | 612 (38.49) | | | 254 (28.57) | | | 358 (51.07) | | |  | |  |
| EBV DNA level after first cycle (%) | | |  | | |  | | |  | | | <0.001 | |
| Undetectable | | 724 (45.53) | | | 724 (81.44) | | | 0 (0.00) | | |  | |  |
| Detectable | | 866 (54.47) | | | 165 (18.56) | | | 701 (100.00) | | |  | |  |
| EBV DNA level after IC (%) | | |  | | |  | | |  | | | <0.001 | |
| Undetectable | | 1073 (67.48) | | | 725 (81.55) | | | 348 (49.64) | | |  | |  |
| Detectable | | 517 (32.52) | | | 164 (18.45) | | | 353 (50.36) | | |  | |  |
| EBV DNA level after CCRT (%) | | |  | | |  | | |  | | | 0.108 | |
| Undetectable | | 1498 (94.21) | | | 861 (96.85) | | | 637 (90.87) | | |  | |  |
| Detectable | | 92 (5.79) | | | 28 (3.15) | | | 64 (9.13) | | |  | |  |
| Concurrent chemotherapy cycles (%) | | |  | | |  | | |  | | | <0.001 | |
| 2 Cycles | | 972 (61.13) | | | 510 (57.37) | | | 462 (65.91) | | |  | |  |
| 3 Cycles | | 618 (38.87) | | | 379 (42.63) | | | 239 (34.09) | | |  | |  |
| Adjuvant therapy (%) | | |  | | |  | | |  | | | <0.001 | |
| No | | 1417 (89.12) | | | 817 (91.90) | | | 600 (85.59) | | |  | |  |
| Yes | | 173 (10.88) | | | 72 (8.10) | | | 101 (14.41) | | |  | |  |

**eTable 2 Baseline characteristics before and after PSM-balancing of** **low-risk group**

|  | Unmatched cohort | | | | | | | | | | PSM-matched cohort | | | | | | | |  |
| --- | --- | --- | --- | --- | --- | --- | --- | --- | --- | --- | --- | --- | --- | --- | --- | --- | --- | --- | --- |
| Characteristics | 2-Cycle | | | | 3-Cycle | | | P values | | | 2-Cycle | | | 3-Cycle | | | P values | |  |
| Overall | 520 | | | | 369 | | |  | | | 247 | | | 247 | | |  | |  |
| Age (%) | |  | | 0.095 | | |  | | |  | | | 0.928 | | |  |  |  |  |
| ≤45 | 237 (45.6) | | | | 190 (51.5) | | |  | | | 125 (50.6) | | | 123 (49.8) | | |  | |  |
| >45 | 283 (54.4) | | | | 179 (48.5) | | |  | | | 122 (49.4) | | | 124 (50.2) | | |  | |  |
| Sex (%) | |  | | 0.502 | | |  | | |  | | | 0.845 | | |  |  |  |  |
| Male | 374 (71.9) | | | | 275 (74.5) | | |  | | | 173 (70.0) | | | 171 (69.2) | | |  | |  |
| Female | 146 (28.1) | | | | 94 (25.5) | | |  | | | 74 (30.0) | | | 76 (30.8) | | |  | |  |
| T stage (%) | |  | | 0.081 | | |  | | |  | | | 0.649 | | |  |  |  |  |
| T1-2 | 33 (6.3) | | | | 36 (9.8) | | |  | | | 22 (8.9) | | | 26 (10.5) | | |  | |  |
| T3-4 | 487 (93.7) | | | | 333 (90.2) | | |  | | | 225 (91.1) | | | 221 (89.5) | | |  | |  |
| N stage (%) | |  | | <0.001 | | |  | | |  | | | 1.00 | | |  |  |  |  |
| N0-1 | 255 (49.0) | | | | 59 (16.0) | | |  | | | 55 (22.3) | | | 56 (22.7) | | |  | |  |
| N2-3 | 265 (51.0) | | | | 310 (84.0) | | |  | | | 192 (77.7) | | | 191 (77.3) | | |  | |  |
| Overall stage (%) | |  | | <0.001 | | |  | | |  | | | 0.562 | | |  |  |  |  |
| III | 400 (76.9) | | | | 230 (62.3) | | |  | | | 172 (69.6) | | | 165 (66.8) | | |  | |  |
| IVA | 120 (23.1) | | | | 139 (37.7) | | |  | | | 75 (30.4) | | | 82 (33.2) | | |  | |  |
| ECOG PS (%) | |  | | 0.066 | | |  | | |  | | | 0.444 | | |  |  |  |  |
| 0-1 | 498 (95.8) | | | | 342 (92.7) | | |  | | | 235 (95.1) | | | 230 (93.1) | | |  | |  |
| > 1 | 22 (4.2) | | | | 27 (7.3) | | |  | | | 12 (4.9) | | | 17 (6.9) | | |  | |  |
| Induction chemotherapy regimen (%) | | |  | | |  | | | <0.001 | | |  | | |  | | | 0.967 | |
| GP | 85 (16.3) | | | | 143 (38.8) | | |  | | | 52 (21.1) | | | 48 (19.4) | | |  | |  |
| TPF | 221 (42.5) | | | | 110 (29.8) | | |  | | | 98 (39.7) | | | 96 (38.9) | | |  | |  |
| TP | 120 (23.1) | | | | 58 (15.7) | | |  | | | 44 (17.8) | | | 50 (20.2) | | |  | |  |
| TPC | 77 (14.8) | | | | 51 (13.8) | | |  | | | 46 (18.6) | | | 46 (18.6) | | |  | |  |
| Other or more | 17 (3.3) | | | | 7 (1.9) | | |  | | | 7 (2.8) | | | 7 (2.8) | | |  | |  |
| Pre-treatment EBV DNA level (%) | | |  | | |  | | | <0.001 | | |  | | |  | | | 0.777 | |
| < 4000 copies/mL | 406 (78.1) | | | | 229 (62.1) | | |  | | | 173 (70.0) | | | 169 (68.4) | | |  | |  |
| ≥ 4000 copies/mL | 114 (21.9) | | | | 140 (37.9) | | |  | | | 74 (30.0) | | | 78 (31.6) | | |  | |  |
| EBV DNA level after first cycle (%) | | |  | | |  | | | <0.001 | | |  | | |  | | | 0.89 | |
| Undetectable | 386 (74.2) | | | | 338 (91.6) | | |  | | | 216 (87.4) | | | 218 (88.3) | | |  | |  |
| Detectable | 134 (25.8) | | | | 31 (8.4) | | |  | | | 31 (12.6) | | | 29 (11.7) | | |  | |  |
| EBV DNA level after IC (%) | | |  | | |  | | | <0.001 | | |  | | |  | | | 0.224 | |
| Undetectable | 394 (75.8) | | | | 331 (89.7) | | |  | | | 201 (81.4) | | | 212 (85.8) | | |  | |  |
| Detectable | 126 (24.2) | | | | 38 (10.3) | | |  | | | 46 (18.6) | | | 35 (14.2) | | |  | |  |
| EBV DNA level after CCRT (%) | | |  | | |  | | | 0.108 | | |  | | |  | | | 1.00 | |
| Undetectable | 499 (96.0) | | | | 362 (98.1) | | |  | | | 241 (97.6) | | | 240 (97.2) | | |  | |  |
| Detectable | 21 (4.0) | | | | 7 (1.9) | | |  | | | 6 (2.4) | | | 7 (2.8) | | |  | |  |
| Concurrent chemotherapy cycles (%) | | |  | | |  | | | <0.001 | | |  | | |  | | | 0.926 | |
| 2 Cycles | 265 (51.0) | | | | 245 (66.4) | | |  | | | 156 (63.2) | | | 154 (62.3) | | |  | |  |
| 3 Cycles | 255 (49.0) | | | | 124 (33.6) | | |  | | | 91 (36.8) | | | 93 (37.7) | | |  | |  |
| Adjuvant therapy (%) | | |  | | |  | | | 0.065 | | |  | | |  | | | 0.612 | |
| No | 470 (90.4) | | | | 347 (94.0) | | |  | | | 226 (91.5) | | | 230 (93.1) | | |  | |  |
| Yes | 50 (9.6) | | | | 22 (6.0) | | |  | | | 21 (8.5) | | | 17 (6.9) | | |  | |  |

**eTable 3 Univariate and multivariate COX regression analysis in low-risk group before PSM-balancing.**

|  | Univariate COX regression | | Multivariate COX regression | |
| --- | --- | --- | --- | --- |
|  | HR (95% CI) | P value | HR (95% CI) | P value |
| Age |  | 0.547 |  |  |
| ≤45 | Reference |  |  |  |
| >45 | 1.12(0.77-1.62) |  |  |  |
| Sex |  | 0.517 |  |  |
| Male | Reference |  |  |  |
| Female | 0.87(0.56-1.34) |  |  |  |
| T Stage |  | 0.922 |  |  |
| T1-2 | Reference |  |  |  |
| T3-4 | 1.03 (0.52-2.04) |  |  |  |
| N Stage |  | 0.605 |  |  |
| N0-1 | Reference |  |  |  |
| N2-3 | 1.12 (0.74-1.69) |  |  |  |
| Overall stage |  | <0.001 |  | <0.001 |
| III | Reference |  |  |  |
| IVA | 1.99 (1.37-2.9) |  | 2.19 (1.50-3.20) |  |
| ECOG PS |  | 0.533 |  |  |
| 0-1 | Reference |  |  |  |
| > 1 | 1.28 (0.59-2.74) |  |  |  |
| Induction chemotherapy regimen |  |  |  |  |
| GP | Reference |  |  |  |
| TPF | 0.71(0.43-1.15) | 0.164 |  |  |
| TP | 0.95(0.55-1.64) | 0.857 |  |  |
| TPC | 0.91(0.46-1.78) | 0.774 |  |  |
| Other or more | 1.28(0.57-2.9) | 0.553 |  |  |
| Induction chemotherapy cycle |  | 0.320 |  |  |
| 2 Cycles | Reference |  |  |  |
| 3 Cycles | 0.83 (0.57-1.2) 0.32 |  |  |  |
| Pre-treatment EBV DNA level |  | 0.181 |  |  |
| < 4000 copies/mL | Reference |  |  |  |
| ≥ 4000 copies/mL | 1.3 (0.89-1.9) |  |  |  |
| EBV DNA level after first cycle |  | 0.422 |  |  |
| Undetectable | Reference |  |  |  |
| Detectable | 1.22 (0.76-1.96) |  |  |  |
| EBV DNA level after IC |  | 0.019 |  | 0.47 |
| Undetectable | Reference |  | Reference |  |
| Detectable | 1.69 (1.09-2.63) |  | 1.33 (0.61-2.95) |  |
| EBV DNA level after CCRT |  | 0.001 |  | 0.002 |
| Undetectable | Reference |  | Reference |  |
| Detectable | 3.6 (1.73-7.49) |  | 3.55(1.60-7.87) |  |
| Concurrent chemotherapy cycles |  | 0.017 |  | 0.003 |
| 2 Cycles | Reference |  | Reference |  |
| 3 Cycles | 0.6 (0.4-0.91) |  | 0.53 (0.35-0.81) |  |
| Adjuvant therapy |  | 0.833 |  |  |
| No | Reference |  |  |  |
| Yes | 0.93 (0.45-1.9) |  |  |  |

**eTable 4 Baseline characteristics before and after PSM-balancing of high-risk group.**

|  | | Unmatched cohort | | | | | | PSM-balancing cohort | | | | | |
| --- | --- | --- | --- | --- | --- | --- | --- | --- | --- | --- | --- | --- | --- |
| Characteristics | | 2-Cycle | | 3-Cycle | | P values | | 2-Cycle | | 3-Cycle | | P values | |
| Overall | | 337 | | 364 | |  | | 213 | | 213 | |  | |
| Age (%) |  | |  | | 0.514 | |  | |  | | 1.000 | |  |
| ≤45 | | 150 (44.5) | | 172 (47.3) | |  | | 101 (47.4) | | 102 (47.9) | |  | |
| >45 | | 187 (55.5) | | 192 (52.7) | |  | | 112 (52.6) | | 111 (52.1) | |  | |
| Sex (%) |  | |  | | 0.901 | |  | |  | | 1.000 | |  |
| Male | | 101 (47.4) | | 102 (47.9) | |  | | 162 (76.1) | | 163 (76.5) | |  | |
| Female | | 112 (52.6) | | 111 (52.1) | |  | | 51 (23.9) | | 50 (23.5) | |  | |
| T (%) |  | |  | | 0.805 | |  | |  | | 1.000 | |  |
| T1-2 | | 40 (11.9) | | 40 (11.0) | |  | | 23 (10.8) | | 22 (10.3) | |  | |
| T3-4 | | 297 (88.1) | | 324 (89.0) | |  | | 190 (89.2) | | 191 (89.7) | |  | |
| N (%) |  | |  | | NA | |  | |  | | NA | |  |
| N0-1 | | 0 (0) | | 0 (0) | |  | | 0 (0) | | 0 (0) | |  | |
| N2-3 | | 337 (100.0) | | 364 (100.0) | |  | | 213 (100.0) | | 213 (100.0) | |  | |
| Stage (%) |  | |  | | 0.224 | |  | |  | | 0.917 | |  |
| III | | 237 (70.3) | | 240 (65.9) | |  | | 146 (68.5) | | 144 (67.6) | |  | |
| IVA | | 100 (29.7) | | 124 (34.1) | |  | | 67 (31.5) | | 69 (32.4) | |  | |
| ECOG PS (%) |  | |  | | 0.04 | |  | |  | | 0.528 | |  |
| 0-1 | | 327 (97.0) | | 340 (93.4) | |  | | 203 (95.3) | | 199 (93.4) | |  | |
| > 1 | | 10 (3.0) | | 24 (6.6) | |  | | 10 (4.7) | | 14 (6.6) | |  | |
| Induction chemotherapy regimen (%) |  | |  | | <0.001 | |  | |  | | 0.958 | |  |
| GP | | 34 (10.1) | | 152 (41.8) | |  | | 34 (16.0) | | 36 (16.9) | |  | |
| TPF | | 142 (42.1) | | 103 (28.3) | |  | | 80 (37.6) | | 82 (38.5) | |  | |
| TP | | 93 (27.6) | | 57 (15.7) | |  | | 53 (24.9) | | 52 (24.4) | |  | |
| TPC | | 53 (15.7) | | 38 (10.4) | |  | | 35 (16.4) | | 30 (14.1) | |  | |
| Other or more | | 15 (4.5) | | 14 (3.8) | |  | | 11 (5.2) | | 13 (6.1) | |  | |
| Pre-treatment EBV DNA level (%) |  | |  | | 0.002 | |  | |  | |  | |  |
| < 4000 copies/mL | | 186 (55.2) | | 157 (43.1) | |  | | 98 (46.0) | | 105 (49.3) | | 0.561 | |
| ≥ 4000 copies/mL | | 151 (44.8) | | 207 (56.9) | |  | | 115 (54.0) | | 108 (50.7) | |  | |
| EBV DNA level after first cycles (%) |  | |  | | NA | |  | |  | | NA | |  |
| Undetectable | | 0 (0) | | 0 (0) | |  | | 0 (0) | | 0 (0) | |  | |
| Detectable | | 337 (100.0) | | 364 (100.0) | |  | | 213 (100.0) | | 213 (100.0) | |  | |
| EBV DNA level after IC (%) |  | |  | | 0.003 | |  | |  | | 1.000 | |  |
| Undetectable | | 147 (43.6) | | 201 (55.2) | |  | | 98 (46.0) | | 97 (45.5) | |  | |
| Detectable | | 190 (56.4) | | 163 (44.8) | |  | | 115 (54.0) | | 116 (54.5) | |  | |
| EBV DNA level after CRT (%) |  | |  | | 0.649 | |  | |  | | 0.633 | |  |
| Undetectable | | 304 (90.2) | | 333 (91.5) | |  | | 193 (90.6) | | 189 (88.7) | |  | |
| Detectable | | 33 (9.8) | | 31 (8.5) | |  | | 20 (9.4) | | 24 (11.3) | |  | |
| Concurrent chemotherapy cycles (%) |  | |  | | 0.170 | |  | |  | | 0.481 | |  |
| 2 Cycles | | 213 (63.2) | | 249 (68.4) | |  | | 139 (65.3) | | 131 (61.5) | |  | |
| 3 Cycles | | 124 (36.8) | | 115 (31.6) | |  | | 74 (34.7) | | 82 (38.5) | |  | |
| Adjuvant therapy (%) |  | |  | | 0.003 | |  | |  | | 0.467 | |  |
| No | | 274 (81.3) | | 326 (89.6) | |  | | 183 (85.9) | | 189 (88.7) | |  | |
| Yes | | 63 (18.7) | | 38 (10.4) | |  | | 30 (14.1) | | 24 (11.3) | |  | |

**eTable 5 Univariate and multivariate COX regression analysis before PSM-balancing in high-risk group.**

|  | Univariate COX regression | | Multivariate COX regression | |
| --- | --- | --- | --- | --- |
|  | HR (95% CI) | P value | HR (95% CI) | P value |
| Age |  | 0.792 |  |  |
| ≤45 | Reference |  |  |  |
| >45 | 1.04(0.77-1.41) |  |  |  |
| Sex |  | 0.823 |  |  |
| Male | Reference |  |  |  |
| Female | 1.04(0.73-1.48) |  |  |  |
| T |  | 0.904 |  |  |
| T1-2 | Reference |  |  |  |
| T3-4 | 1.03(0.65-1.64) |  |  |  |
| N |  | NA |  |  |
| N0-1 | Reference |  |  |  |
| N2-3 | / |  |  |  |
| Stage |  | 0.812 |  |  |
| III | Reference |  |  |  |
| IVA | 0.96(0.7-1.33) |  |  |  |
| ECOG PS |  | 0.265 |  |  |
| 0-1 | Reference |  |  |  |
| > 1 | 1.44(0.76-2.73) |  |  |  |
| Induction chemotherapy regimen |  |  |  |  |
| GP | Reference |  |  |  |
| TPF | 1.1(0.73-1.65) | 0.659 |  |  |
| TP | 1.37(0.87-2.16) | 0.169 |  |  |
| TPC | 1.25(0.71-2.21) | 0.437 |  |  |
| Other or more | 1.71(0.89-3.29) | 0.109 |  |  |
| IC cycle |  | 0.963 |  |  |
| 2 Cycles | Reference |  |  |  |
| 3 Cycles | 0.99(0.74-1.34) |  |  |  |
| Pre-treatment EBV DNA level |  | 0.014 |  | 0.074 |
| < 4000 copies/mL | Reference |  | Reference |  |
| ≥ 4000 copies/mL | 1.47(1.08-1.99) |  | 1.32 (0.97-1.81) |  |
| EBV DNA level after first cycles |  | NA |  |  |
| Undetectable | Reference |  |  |  |
| Detectable | / |  |  |  |
| EBV DNA level after IC |  | <0.001 |  | <0.001 |
| Undetectable | Reference |  | Reference |  |
| Detectable | 1.89(1.39-2.57) |  | 1.85 (1.34-2.56) |  |
| EBV DNA level after CRT |  | <0.001 |  | 0.001 |
| Undetectable | Reference |  | Reference |  |
| Detectable | 2.81(1.81-4.34) |  | 2.14 (1.36-3.35) |  |
| Concurrent chemotherapy cycles |  | 0.024 |  | 0.005 |
| 2 Cycles | Reference |  | Reference |  |
| 3 Cycles | 0.68 (0.48-0.95) |  | 0.61 (0.43-0.86) |  |
| Adjuvant therapy |  | 0.440 |  |  |
| No | Reference |  |  |  |
| Yes | 0.83(0.51-1.34) |  |  |  |

**Supplementary Figures**


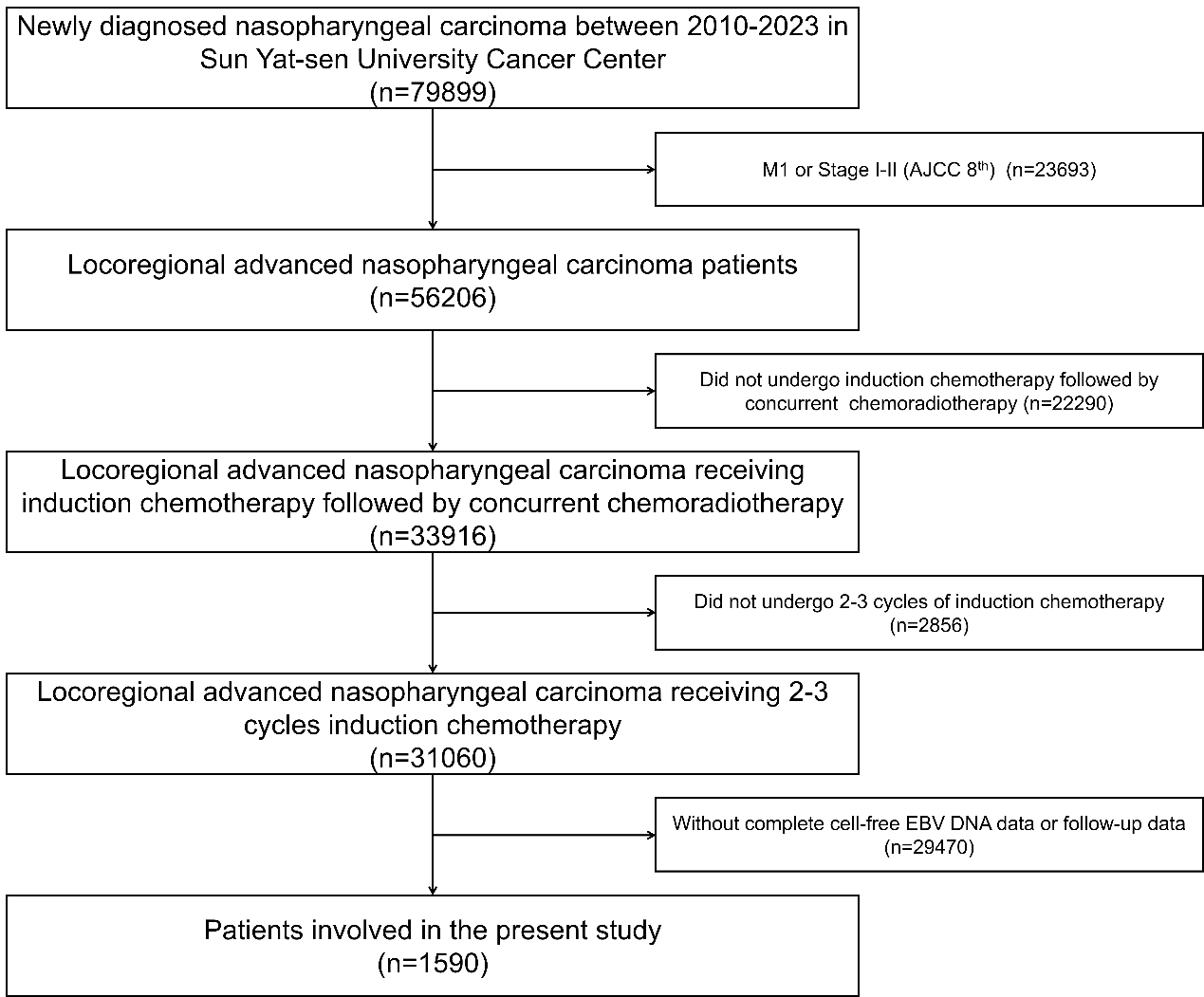


**eFigure 1. Flowchart of the present study.**

A total of 79,899 newly diagnosed nasopharyngeal carcinoma (NPC) patients at Sun Yat-sen University Cancer Center (2010–2023) were screened. Exclusions included: M1 disease or stage I–II NPC (n=23,693), no induction chemotherapy (IC) followed by concurrent chemoradiotherapy (CCRT) (n=22,290), incomplete 2–3 cycles of IC (n=2,856), and missing cell-free EBV (cfEBV) DNA data or insufficient follow-up (n=29,470). Finally, 1,590 locoregionally advanced NPC patients who received IC+CCRT were included in the analysis.


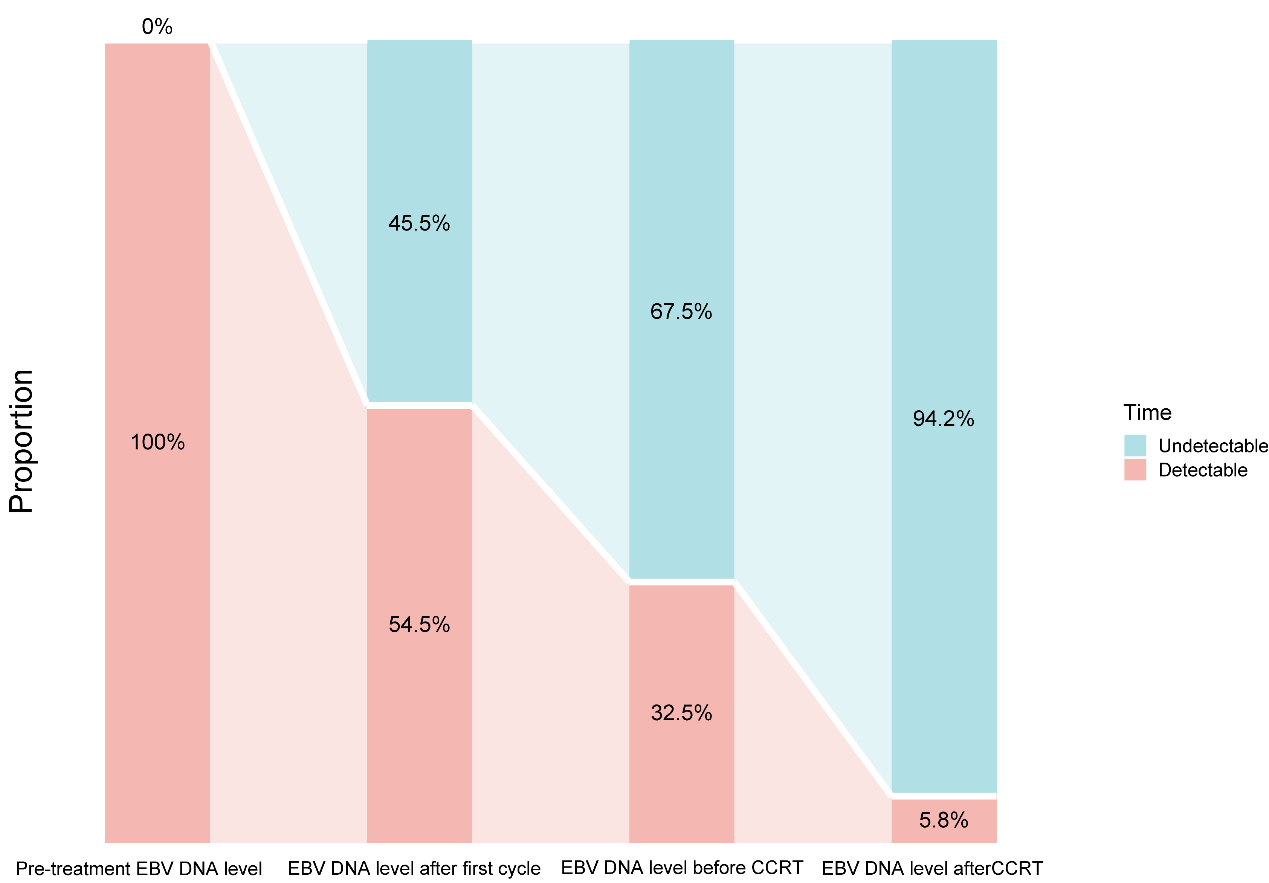


**eFigure 2. Dynamic flow of cfEBV DNA detectability across critical treatment Nodes in locoregionally advanced nasopharyngeal carcinoma patients.**

Sankey diagram depicting the proportion flow of detectable vs. undetectable cell-free Epstein-Barr virus (EBV) DNA levels across four key treatment time points in the whole cohort of 1,590 locoregionally advanced nasopharyngeal carcinoma (LA-NPC) patients.


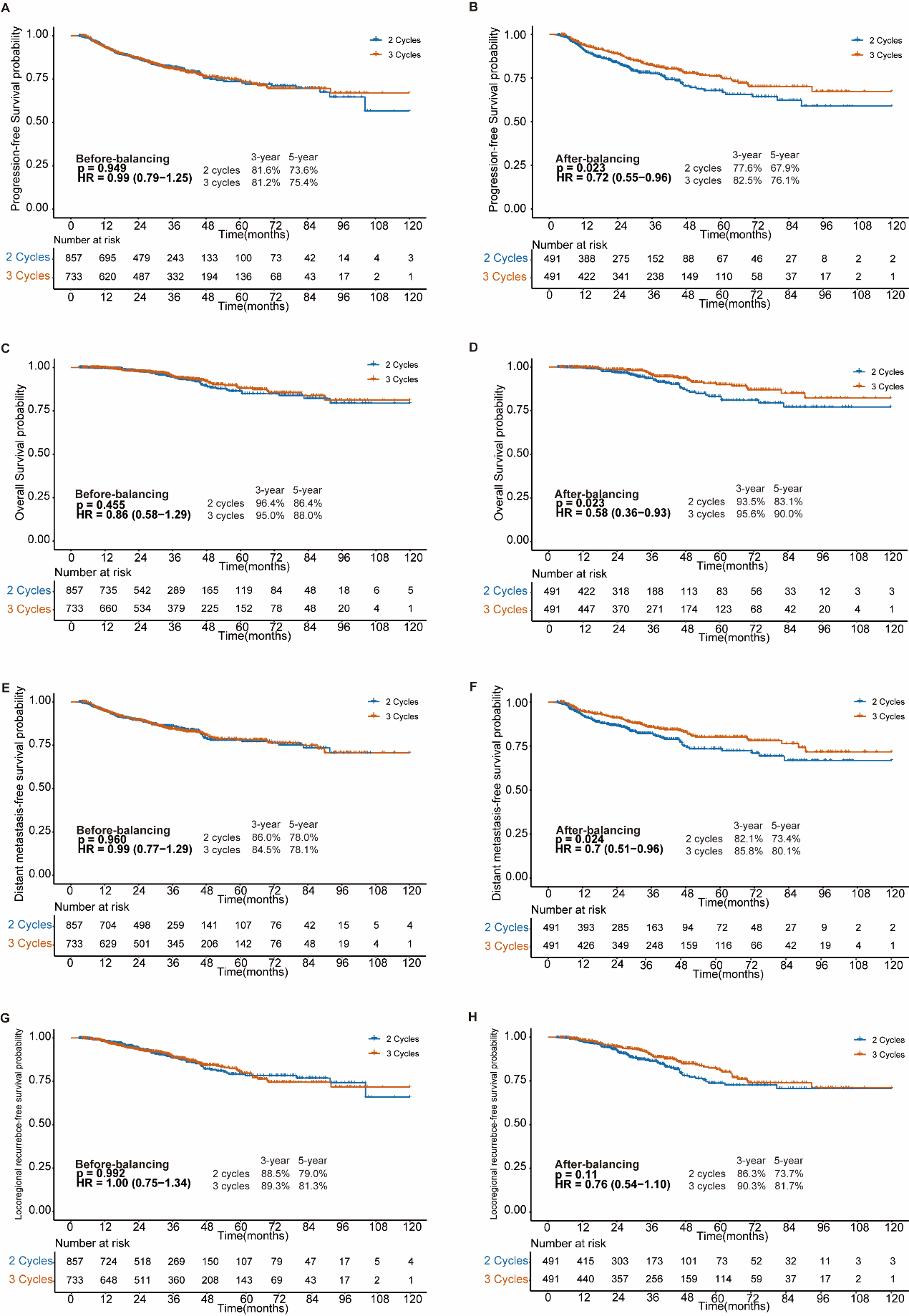


**eFigure 3. Survival outcomes comparison between 2-cycle and 3-cycle IC of the whole cohort before and after PSM.** (A) Progression-free survival before PSM; (B) Progression-free survival after PSM; (C) Overall survival before PSM; (D) Overall survival after PSM; (E) Distant metastasis-free survival before PSM; (F) Distant metastasis-free survival after PSM; (G) Locoregional recurrence-free survival before PSM; (H) Locoregional recurrence-free survival after PSM.
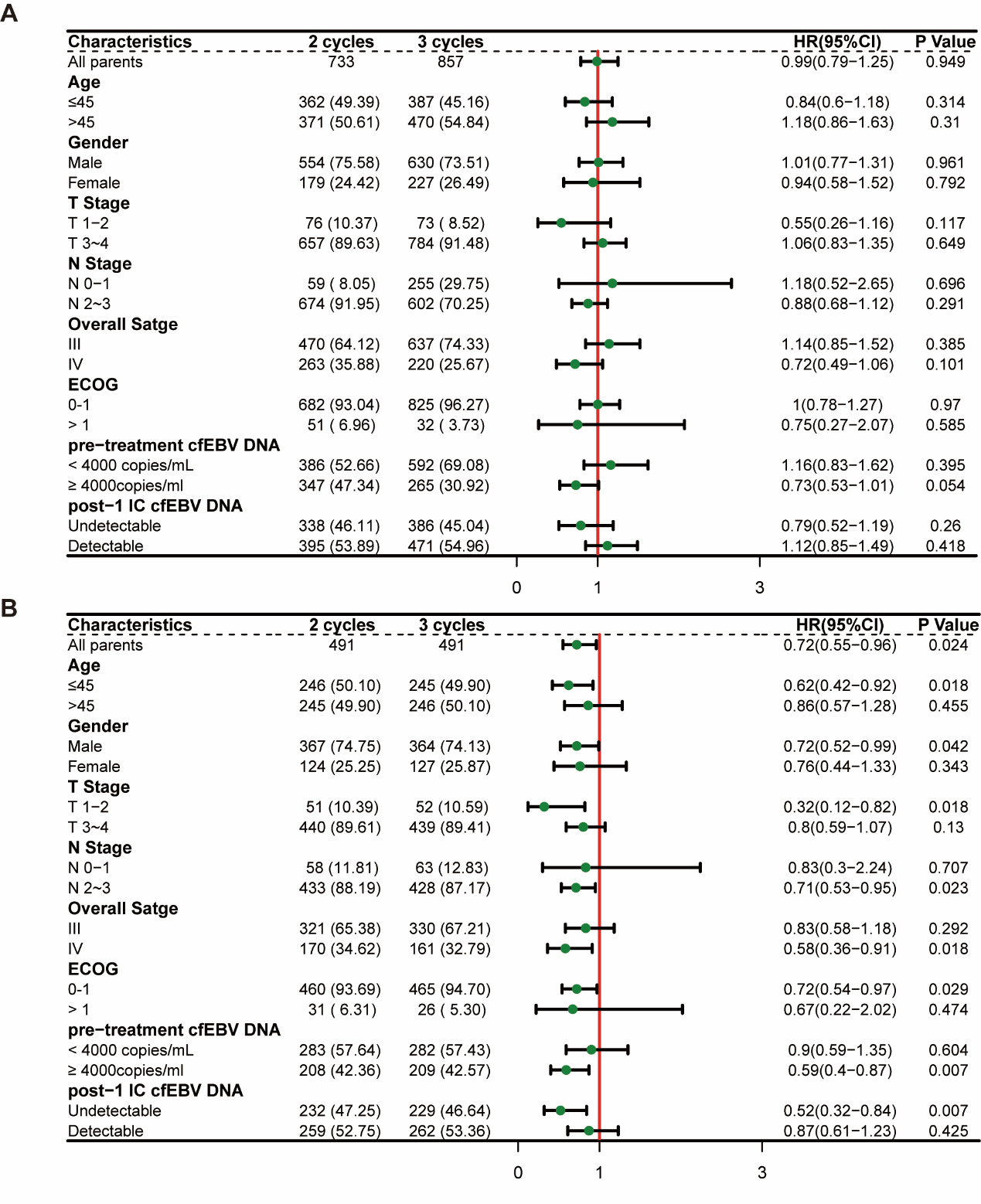
 **eFigure 4. Subgroup analyses for progression-free survival of the whole cohort before and after PSM.** (A) Subgroup analysis before PSM, (B) Subgroup analysis after PSM.


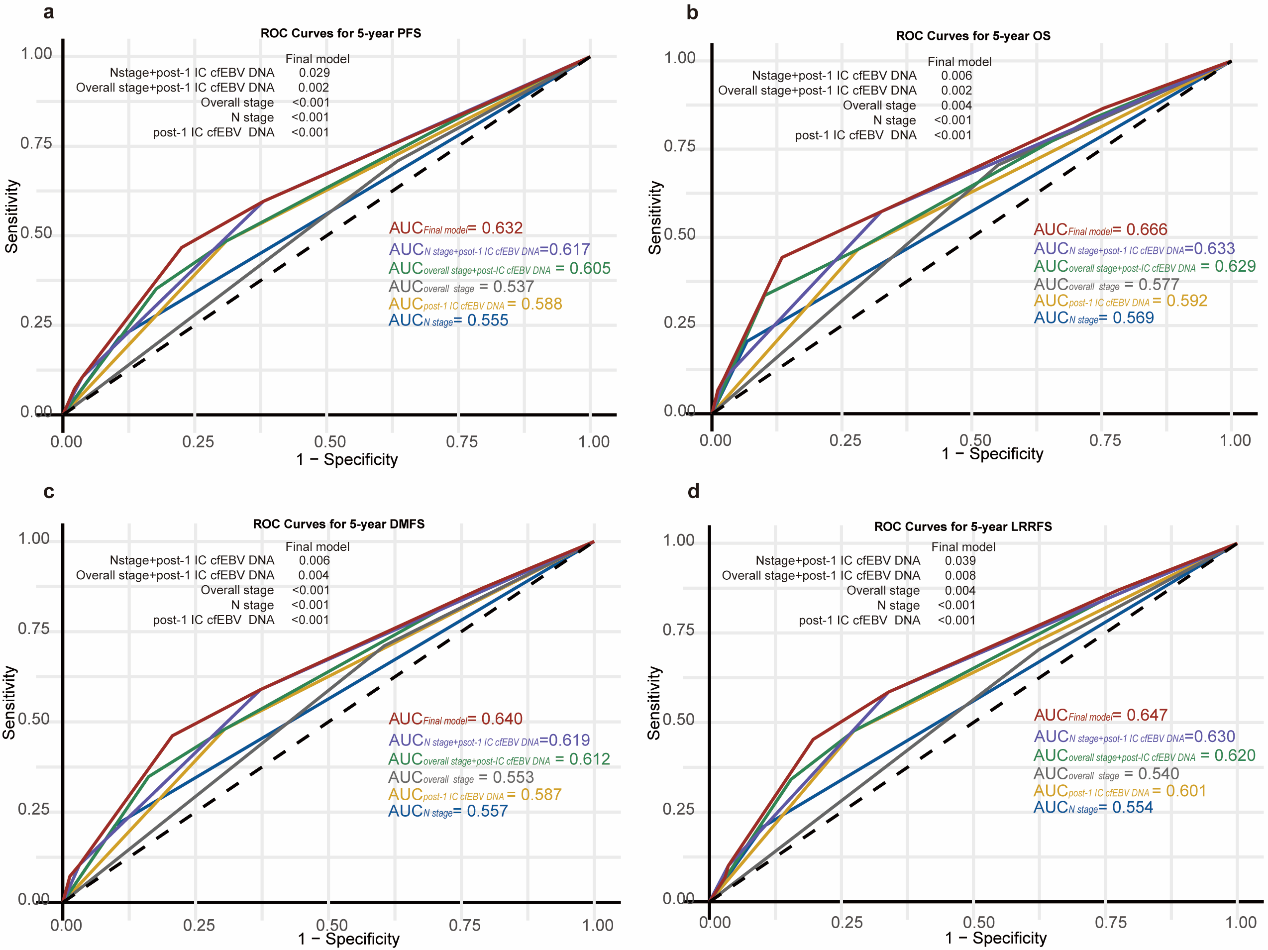


**eFigure 5. 5-year receiver operating characteristic curves for univariate (N stage/Overall Stage/post-1 IC cfEBV DNA), bivariate (N stage+post-1 IC cfEBV DNA/Overall stage+post-1 IC cfEBV DNA), and full-variable (N stage+Overall Stage+post-1 IC cfEBV DNA) analyses.** (A) Progression-free survival; (B) Overall survival; (C) Distant metastasis-free survival; (D) Locoregional recurrence-free survival.


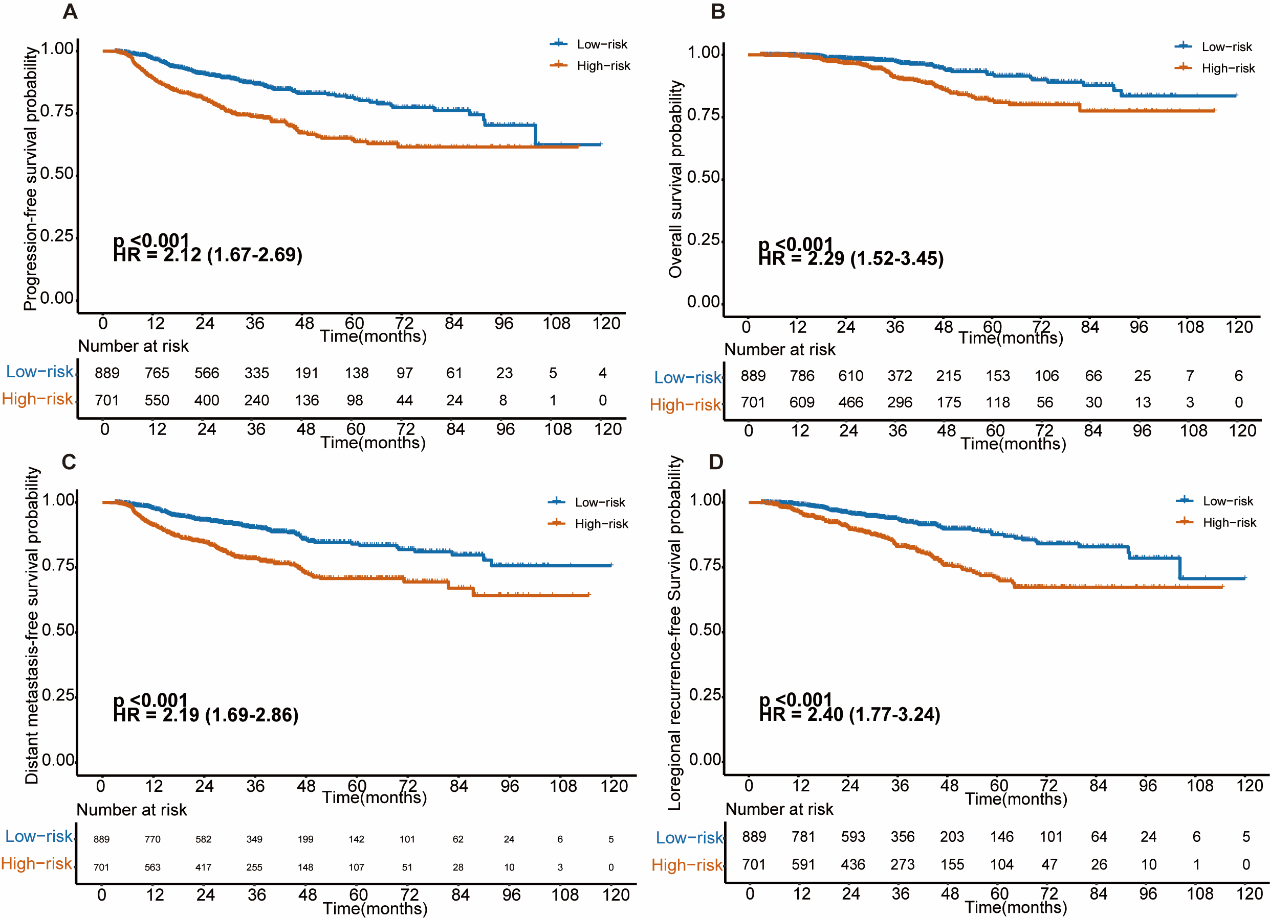


**eFigure 6. Survival outcomes comparison between high- and low-risk groups.** (A) Progression-free survival; (B) Overall survival; (C) Distant metastasis-free survival; (D) Locoregional recurrence-free survival.


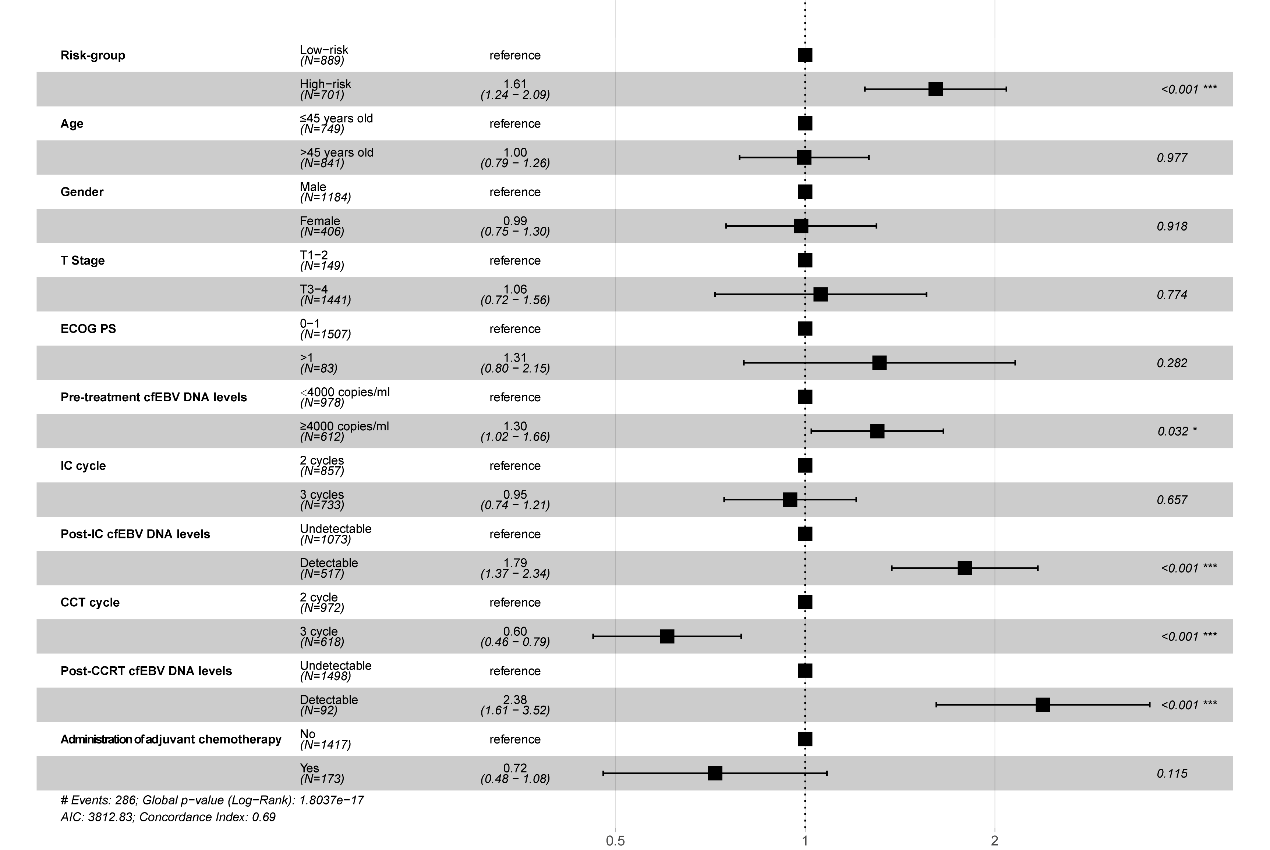


**eFigure 7. Forest plot of multivariate Cox proportional hazards regression analysis for progression-free survival (PFS).**


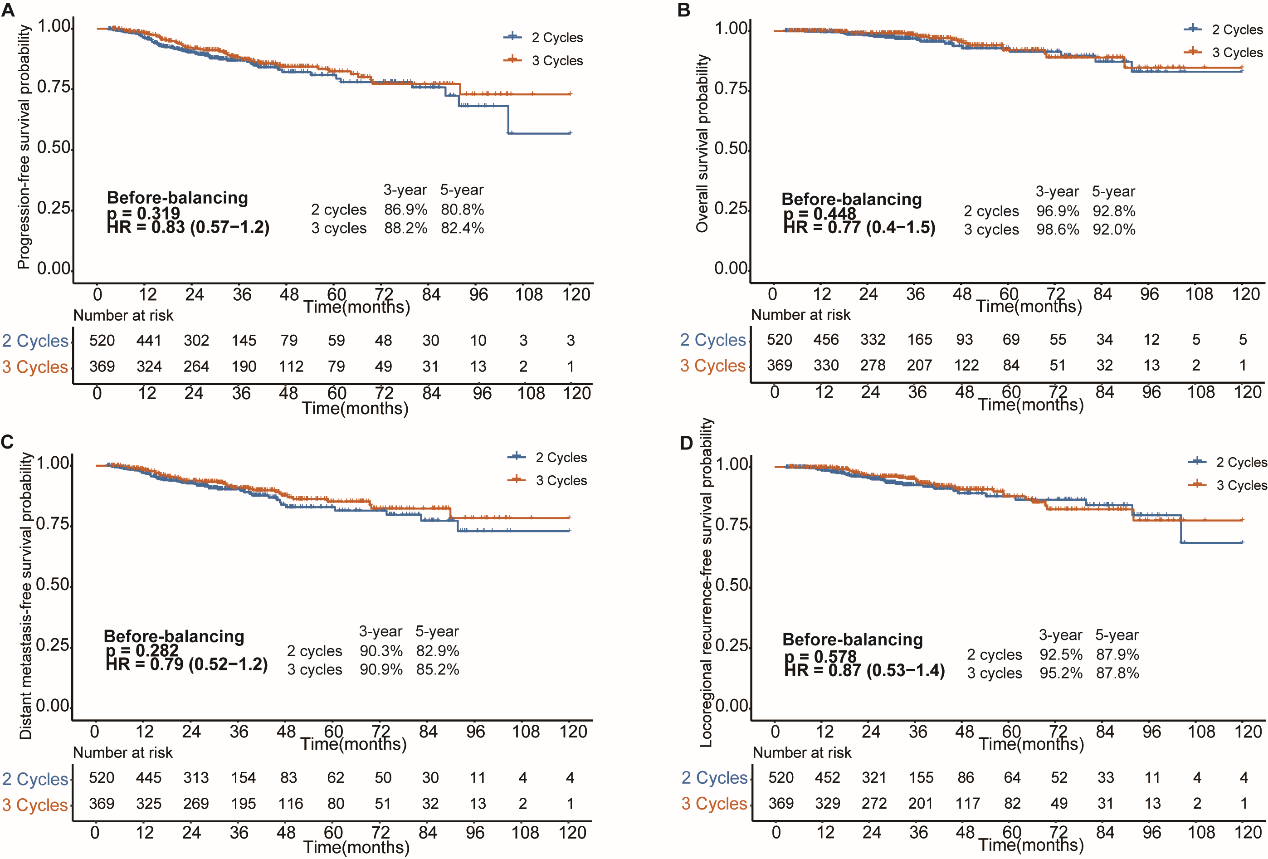


**Figure 8. Survival outcomes comparison between 2-cycle and 3-cycle IC of the low-risk group before PSM.** (A) Progression-free survival before PSM; (B) Overall survival before PSM; (C) Distant metastasis-free survival before PSM; (D) Locoregional recurrence-free survival before PSM.


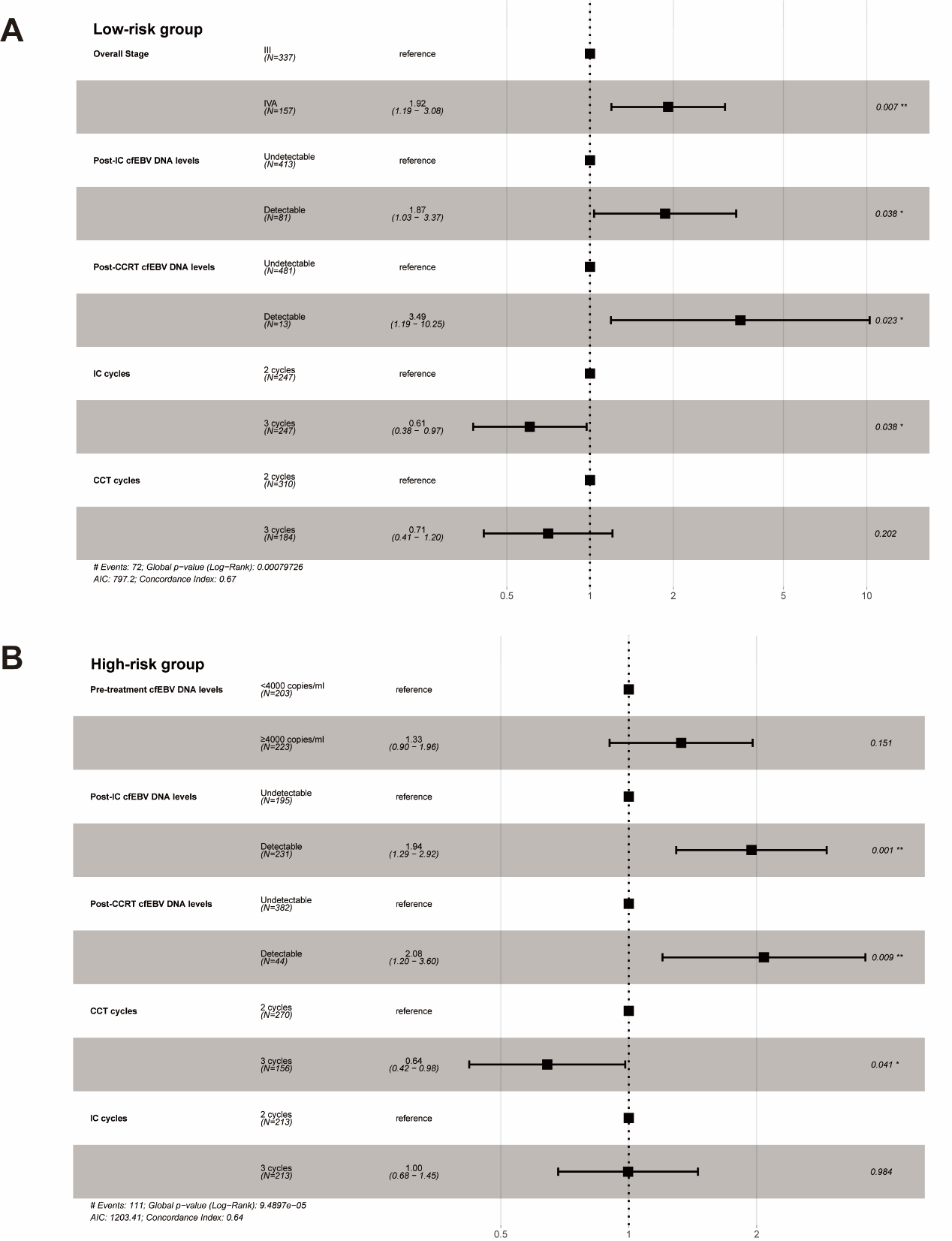


**eFigure 9. Forest plot of multivariate Cox proportional hazards regression analysis for progression-free survival (PFS) of low- and high- risk groups.** (A) Low-risk group, (B) High-risk group.


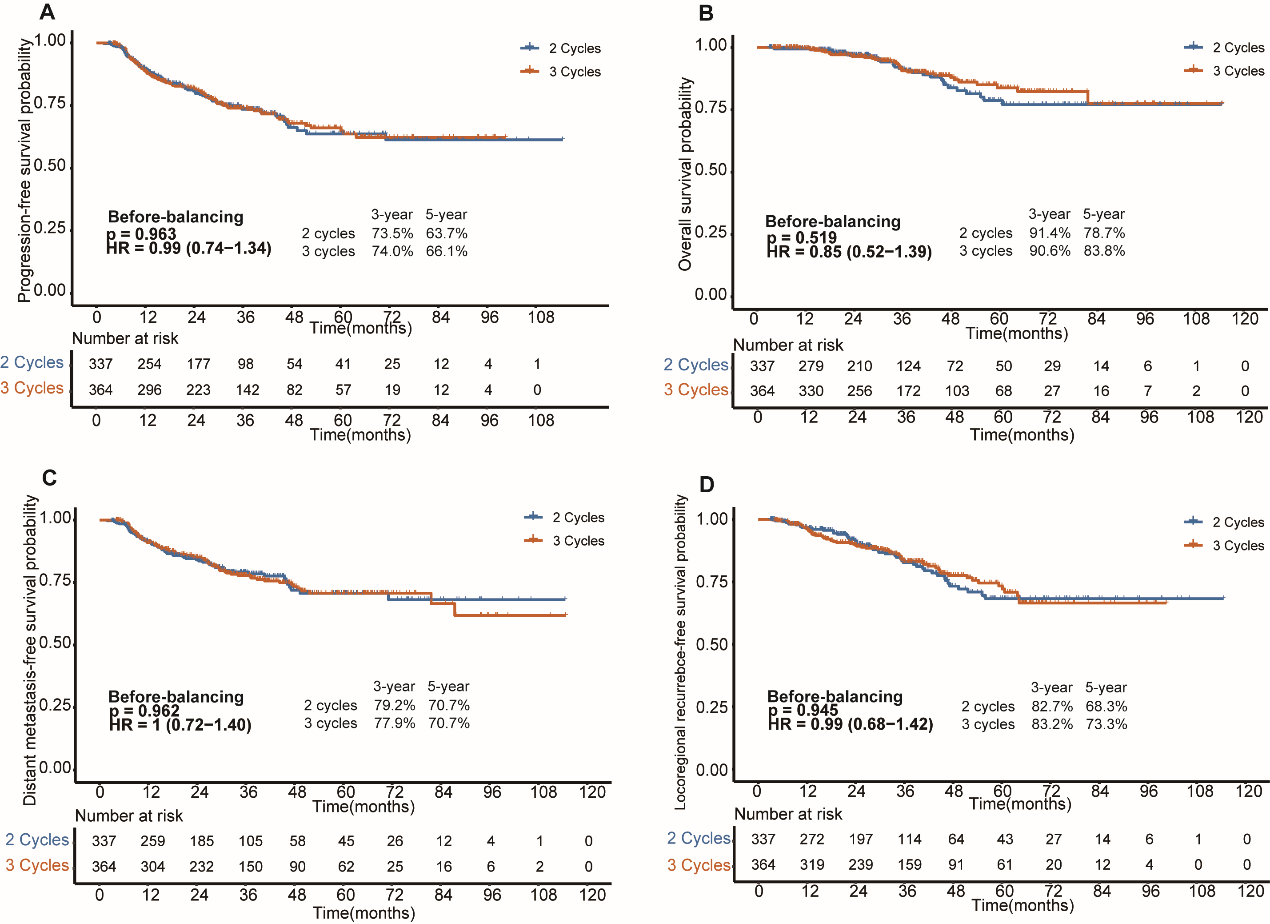


**Figure 10. Survival outcomes comparison between 2-cycle and 3-cycle IC of the High-risk group before PSM.** (A) Progression-free survival before PSM; (B) Overall survival before PSM; (C) Distant metastasis-free survival before PSM; (D) Locoregional recurrence-free survival before PSM.
